# ﻿*Rostrupomyces*, a new genus to accommodate *Xerocomussisongkhramensis*, and a new *Hemileccinum* species (Xerocomoideae, Boletaceae) from Thailand

**DOI:** 10.3897/mycokeys.103.107935

**Published:** 2024-03-28

**Authors:** Santhiti Vadthanarat, Bhavesh Raghoonundon, Saisamorn Lumyong, Olivier Raspé

**Affiliations:** 1 School of Science, Mae Fah Luang University, Chiang Rai, 57100, Thailand; 2 Department of Biological Science, Faculty of Science, Ubon Ratchathani University, Ubon Ratchathani, 34190, Thailand; 3 Department of Biology, Faculty of Science, Chiang Mai University, Chiang Mai, 50200, Thailand; 4 Research Center of Microbial Diversity and Sustainable Utilization, Faculty of Science, Chiang Mai University, Chiang Mai, 50200, Thailand; 5 Academy of Science, The Royal Society of Thailand, Bangkok, Thailand; 6 Meise Botanic Garden, Nieuwelaan 38, 1860 Meise, Belgium; 7 Service Général de l’Enseignement Supérieur et de la Recherche Scientifique, Fédération Wallonie-Bruxelles, Brussels, Belgium

**Keywords:** *atp*6, Boletales, *cox*3, fungal diversity, multigene phylogeny, one new species, taxonomy, Tropical Asia

## Abstract

A new genus, *Rostrupomyces* is established to accommodate *Xerocomussisongkhramensis* based on multiple protein-coding genes (*atp*6, *cox*3, *tef*1, and *rpb*2) analyses of a wide taxon sampling of Boletaceae. In our phylogeny, the new genus was sister to *Rubinosporus* in subfamily Xerocomoideae, phylogenetically distant from *Xerocomus*, which was highly supported as sister to *Phylloporus* in the same subfamily Xerocomoideae. *Rostrupomyces* is different from other genera in Boletaceae by the following combination of characters: rugulose to subrugulose pileus surface, white pores when young becoming pale yellow in age, subscabrous stipe surface scattered with granulose squamules, white basal mycelium, unchanging color in any parts, yellowish brown spore print, and broadly ellipsoid to ellipsoid, smooth basidiospores. In addition, *Hemileccinuminferius*, also from subfamily Xerocomoideae, is newly described. Detailed descriptions and illustrations of the new genus and new species are presented.

## ﻿Introduction

Xerocomoideae Singer, which is one of the six subfamilies in Boletaceae Chevall, was established in 1945 with *Xerocomus* Quél. as the typus. At present, the subfamily consists of 12 genera, namely *Alessioporus* Gelardi, Vizzini & Simonini, *Amylotrama* Bloomfield, Davoodian, Trappe & T. Lebel, *Aureoboletus* Pouzar, *Boletellus* Murrill, *Heimioporus* E. Horak, *Hemileccinum* Šutara, *Hourangia* Xue T. Zhu & Zhu L. Yang, *Phylloporus* Quél., *Pulchroboletus* Gelardi, Vizzini & Simonini, *Rubinosporus* Vadthanarat, Raspé & Lumyong, *Veloboletus* Fechner & Halling, and *Xerocomus* (Šutara 2008; Gelardi et al. 2014; Wu et al. 2014; Zhu et al. 2015; Wu et al. 2016; Crous et al. 2020; Lebel et al. 2022; Vadthanarat et al. 2022). The typical characters of species in this subfamily are boletoid or phylloporoid, rarely sequestrate basidiomata; dry or viscid pileus with smooth or subtomentose to tomentose pellis; absence or rarely presence of a veil; off-white, yellowish white, yellowish to yellow context; at least some basidiome parts often bluing, sometimes reddening or unchanging; smooth or ornamented stipe surface; hymenophore yellowish to yellow to bright yellow or cream to dull yellow to yellow to gray in sequestrate forms; basidiospores with bacillate, reticulate, tiny warts, pinholes, longitudinally striate, pitted ornamentations, or occasionally smooth; spore deposit with more or less olive-brown tint, rarely dark ruby (e. g. Gelardi et al. 2014; Wu et al. 2014; Zhu et al. 2015; Wu et al. 2016; Crous et al. 2020; Lebel et al. 2022; Vadthanarat et al. 2022).

*Hemileccinum*, one of the genera belonging to the Xerocomoideae, was established in 2008 to accommodate two *Boletus* species, namely *B.depilatus* Redeuilh and *B.impolitus* Fr. In 2012, a new genus named *Corneroboletus* N.K. Zeng & Zhu L. Yang was established to accommodate *Boletusindecorus* Massee (Zeng et al. 2012). However, *Corneroboletus* was later synonymized with *Hemileccinum* (Wu et al. 2016). *Hemileccinum* currently comprises 13 species worldwide, namely *H.albidum* Mei Xiang Li, Zhu L. Yang & G. Wu, *H.brevisporum* Mei Xiang Li, Zhu L. Yang & G. Wu, *H.brunneotomentosum* (B. Ortiz) Nitson & J.L. Frank, *H.depilatum* (Redeuilh) Šutara, *H.ferrugineipes* Mei Xiang Li, Zhu L. Yang & G. Wu, *H.floridanum* J.A. Bolin, A.E. Bessette, A.R. Bessette, L.V. Kudzma, A. Farid & J.L. Frank, *H.hortonii* (A.H. Sm. & Thiers) M. Kuo & B. Ortiz, *H.impolitum* (Fr.) Šutara (typus), *H.indecorum* (Massee) G. Wu & Zhu L. Yang, *H.parvum* Mei Xiang Li, Zhu L. Yang & G. Wu, *H.rubropunctum* (Peck) Halling & B. Ortiz, *H.rugosum* G. Wu & Zhu L. Yang, *H.subglabripes* (Peck) Halling (Index Fungorum, accessed on 23 March 2023). *Hemileccinum* species share the following combination of characters: boletoid basidiomata, glabrous to subtomentose, smooth to rugose pileus surface, which turns violet with NH_3_ vapours; tubes depressed around the stipe apex, pores at first light yellow to deep yellow becoming olive-yellow in age, concolorous with tubes, unchanging; olive spore deposit; central stipe, whose surface is always ornamented with scales concolorous with stipe, unchanging; pale yellow to light yellow context, unchanging; pileipellis a trichodermium with broad hyphae or an epithelium, sometime with filamentous terminal elements; pleurocystidia present, fusoid to lageniform; spores boletoid, subfusoid or ellipsoid in face view, smooth under light microscope, irregularly tiny warted and pinholed or rarely smooth under SEM; clamp connections absent (Šutara 2008; Halling et al. 2015; Wu et al. 2016; Index Fungorum 443:1, 2020; Kuo and Ortiz-Santana 2020; Farid et al. 2021; Li et al. 2021).

The first study of poroid mushrooms from Thailand was published in 1902, with descriptions of five new species, namely *Boletuslacunosus* Rostr. [current name: *Austroboletusrostrupii* (Syd. & P. Syd.) E. Horak], *Boletuscostatus* Rostr., *Suilluschangensis* Rostr. [current name: *Boletuschangensis* (Rostr.) Sacc. & D. Sacc.], *Suillushygrophanus* Rostr. [current name: *Boletushygrophanus* (Rostr.) Sacc. & D. Sacc.], and *Suillusvelatus* Rostr. [current name: *Veloporphyrellusvelatus* (Rostr.) Y.C. Li & Zhu L. Yang] (Rostrup 1902; Saccardo and Saccardo 1905; Horak 1980; Li et al. 2014). At that time, they were classified to belong to the Polyporaceae; however, later they were all moved to family Boletaceae. No new taxa in Boletaceae were described from Thailand during the following one hundred years. It is only in 2006 that again a new species, *Rhodactinaincarnata* Zhu L. Yang, Trappe & Lumyong, was described from Chiang Mai Province, northern Thailand (Yang et al. 2006). In 2009, *Spongiformathailandica* Desjardin, Manfr. Binder, Roekring & Flegel was described as a new genus and species from Nakorn Nayok Province, central Thailand (Desjardin et al. 2009). After that, molecular phylogenetic analyses have been widely used in Boletaceae taxonomy. Two more new Boletaceae genera including *Cacaoporus* Raspé & Vadthanarat and *Rubinosporus* Vadthanarat, Raspé & Lumyong were described from Chiang Mai Province, northern Thailand (Vadthanarat et al. 2019b, 2022). During that period, twenty-seven new species were also described from the country, among which nine belong in subfamily Xerocomoideae, namely *Heimioporussubcostatus* Vadthanarat, Raspé & Lumyong, *Phylloporuscastanopsidis* M.A. Neves & Halling, *P.dimorphus* M.A. Neves & Halling, *P.infuscatus* M.A. Neves & Halling, *Phylloporuspusillus* Raspé, K.D. Hyde & Chuankid, *P.rubiginosus* M.A. Neves & Halling, *P.subrubeolus* Chuankid, K.D. Hyde & Raspé, *Rubinosporusauriporus* Vadthanarat, Raspé & Lumyong, *Xerocomussisongkhramensis* Khamsuntorn, Pinruan & Luangsa-ard (Neves et al. 2012; Halling et al. 2014; Raspé et al. 2016; Vadthanarat et al. 2018; Chuankid et al. 2019; Vadthanarat et al. 2019a, 2019b, 2020; Chuankid et al. 2021; Raghoonundon et al. 2021; Vadthanarat et al. 2021; Tan et al. 2022; Vadthanarat et al. 2022).

In this study, several collections of boletes belonging to the subfamily Xerocomoideae were obtained from northern and northeastern Thailand. They were carefully studied based on morphology as well as family-wide and subfamily-wide phylogenetic analyses. Some of them were identified as a new *Hemileccinum* species. Some collections were identified as *X.sisongkhramensis* based on morphological characters and the megablast result of the ITS region. However, following multiple gene phylogenetic analyses based on four protein-coding gene (*atp*6, *cox*3, *tef*1, and *rpb*2), *X.sisongkhramensis* appeared phylogenetically distant from other *Xerocomus* species and distinct from existing genera in Boletaceae. Moreover, the detailed morphology did not fit any known Xerocomoideae genus. Therefore, *Rostrupomyces* is introduced to accommodate *X.sisongkhramensis*. Finally, a new *Hemileccinum* species is introduced with full descriptions and illustrations.

## ﻿Materials and methods

### ﻿Specimens collecting

Fresh basidiomata of boletes in subfamily *Xerocomoideae* were collected in Chiang Mai and Chiang Rai provinces in northern Thailand, and Ubon Ratchathani and Sisaket provinces in northeastern Thailand between 2015 and 2021. They were photographed in the field and then wrapped in aluminum foil for later description in the laboratory on the same day. The specimens were then dried in an electric drier at 45–50 °C. Examined specimens were deposited at MFU, BKF or CMUB herbaria.

### ﻿Morphological study

Macroscopic descriptions were made based on the detailed field notes and photos of fresh basidiomata. Color codes were given based on Kornerup and Wanscher (1978). Macrochemical reactions (color reactions) were observed using aqueous solutions of 10% potassium hydroxide (KOH), and 28–30% NH_4_OH. Microscopic structures were observed from dried specimens rehydrated in 5% KOH or 1% ammoniacal Congo red. For the measurements of microscopic features, a minimum of 50 basidiospores or 20 for other structures, were randomly chosen and measured under a Nikon Eclipse Ni compound microscope using NIS-Elements D version 5.10 software. The notation ‘[x/y/z]’ represents the number of basidiospores ‘x’ measured from the number of basidiomata ‘y’ of the number of collections ‘z’. The measurements of microscopic structures are presented in the following format (a–) b–c–d (–e), in which ‘c’ represents the average, ‘b’ is the 5^th^ percentile, ‘d’ is the 95^th^ percentile, and ‘a’ and ‘e’ the extreme values, shown in parentheses. *Q* is the length/width ratio. Sections of the pileipellis were cut radially, perpendicularly to the surface halfway between the centre and margin of pileus. Sections of stipitipellis were taken halfway along the stipe length (Li et al. 2011; Hosen et al. 2013; Li et al. 2014; Zhu et al. 2015). All line drawings of microscopic features were drawn by free hand using an Olympus compound microscope model CX41 with Olympus Camera Lucida model U−DA. For scanning electron microscopy, small fragments of dried hymenophore were mounted directly onto a SEM stub with double-sided carbon tape. The samples were coated with gold, examined and photographed using a TESCAN MIRA’s 4^th^ generation SEM.

### ﻿DNA extraction, PCR amplification and DNA sequencing

Genomic DNA was extracted from tissue of dried specimen or fresh tissue preserved in CTAB, using a CTAB isolation procedure adapted from Doyle and Doyle (1990). Portions of the genes *atp*6, *cox*3, *rpb*2, and *tef*1 were amplified by polymerase chain reaction (PCR). The primer pairs ATP6-1M40F/ATP6-2M (Raspé et al. 2016), COX3M1-F/ COX3M1-R (Vadthanarat et al. 2019b), bRPB2-6F/bRPB2-7.1R (Matheny 2005), and EF1-983F/EF1-2218R (Rehner and Buckley 2005) were used to amplify *atp*6, *cox*3, *rpb*2, and *tef*1, respectively. PCR products were purified by adding 1 U of exonuclease I and 0.5 U FastAP alkaline phosphatase (Thermo Scientific, St. Leon-Rot, Germany) and incubated at 37 °C for 1 h, followed by inactivation at 80 °C for 15 min. Standard Sanger sequencing was performed in both directions by Macrogen with PCR primers, except for *atp*6, for which universal primers M13F-pUC(-40) and M13F(-20) were used. For *tef*1, additional sequencing was performed with two internal primers, EF1-1577F and EF1-1567R (Rehner and Buckley 2005).

### ﻿Alignment and phylogeny inference

The two reads of newly generated sequences were assembled in GENEIOUS Pro v. 6.0.6 (Biomatters) and blasted against GenBank database to check that they were not from unrelated contamination. For the Boletaceae-wide tree, the introns in *rpb*2 and *tef*1 were removed based on the amino acid sequence of previously published sequences. The sequence datasets including the newly generated sequences and selected sequences representative of the whole family downloaded from GenBank, were separately aligned for each gene using MAFFT on the server accessed at http://mafft.cbrc.jp/alignment/server/ (Katoh and Standley 2013). Before combining the four gene partitions (*atp*6, *cox*3, *rpb*2 exons + *tef*1 exons), topological incongruence between the datasets was assessed using maximum likelihood (ML) on each of mitochondrial genes (*atp*6 + *cox*3) dataset and nuclear genes (*rpb*2 exons + *tef*1 exons) dataset. Paired trees were examined for conflicts involving only nodes with ML bootstrap (BS) ≥ 70%. After that, the Maximum likelihood phylogenetic inference was performed using RAxML (Stamatakis 2006) on the CIPRES web portal (RAxML-HPC2 on XSEDE; Miller et al. 2009). The phylogenetic tree was inferred by a single partitioned analysis with four character sets (one for each gene), using the GTRCAT model with 25 categories. The outgroup consisted of two *Buchwaldoboletus* and seven *Chalciporus* species from subfamily Chalciporoideae, based on previously published phylogenies. Statistical support of clades was obtained with 1,000 rapid bootstrap replicates. For Bayesian Inference (BI), the best-fit model of substitution among those implementable in MrBayes was estimated separately for each region using jModel-test (Darriba et al. 2012) on the CIPRES portal, based on the Bayesian Information Criterion (BIC). The selected models were HKY+I+G for *atp*6, GTR+I+G for *cox*3, K80+I+G for *rpb*2 exons, and SYM+I+G for *tef*1 exons. Partitioned Bayesian analysis was performed on the CIPRES web portal (MrBayes on XSEDE; Ronquist et al. 2012). Two runs of five chains were run for 15,000,000 generations and sampled every 1,000 generations. At the end of the run, the average deviation of split frequencies was 0.008563. The PSRF values were equal or greater than 1, and ESS values were greater than 200 for all parameters. A total of 11,252 trees were used to construct a 50% majority rule consensus tree and calculate the Bayesian posterior probabilities (BPPs).

A second, Xerocomoideae-wide tree, was also inferred from sequences of selected taxa in Xerocomoideae. Sequences were also separately aligned for each of the genes using the MAFFT online software, with introns included. Then, the topological incongruence between the datasets was also assessed using ML on each gene of five character sets, *atp*6, *cox*3, *rpb*2 exons, *tef*1 exons, and the three introns of *tef*1 + an intron of *rpb*2. Since there was no supported conflict, the ML phylogenetic tree was inferred by a single partitioned analysis with the five character sets (*atp*6, *cox*3, *rpb*2 exons, *tef*1 exons, and *rpb*2 intron + *tef*1 introns), using the same software and model that was used for family Boletaceae-wide phylogeny. Based on the latter, three *Hourangia*, three *Phylloporus*, and three *Xerocomus* species in the same subfamily Xerocomoideae were used as the outgroup. For BI, partitioned Bayesian analysis was performed with MrBayes 3.2.6 software for Windows. The selected models were GTR+I+G for *atp*6 and *cox*3, K80+I+G for *rpb*2 exons, and SYM+I+G for *tef*1 exons, HKY+I+G intron of *rpb*2 + introns of *tef*1. Two runs of five chains were sampled every 200 generations and stopped after 700,000 generations. At the end of the run, the average deviation of split frequencies was 0.007178. The PSRF values were equal or greater than 1, and ESS values were greater than 200 for all parameters. A total of 2,495 trees were used to construct a 50% majority rule consensus tree and calculate the BPPs.

## ﻿Results

### ﻿Phylogenetic analyses

**Figure 1.**
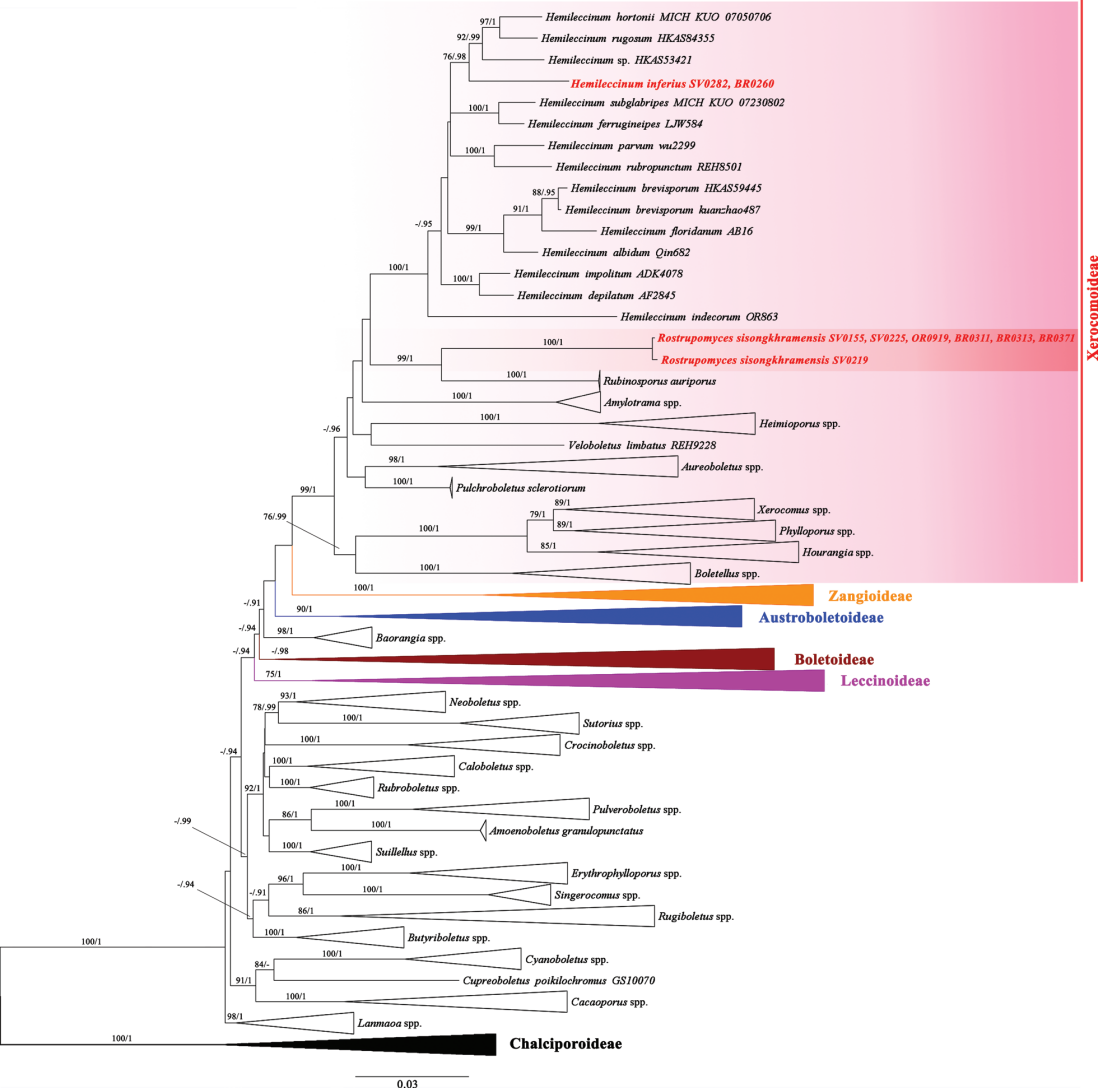
Boletaceae-wide Maximum Likelihood phylogenetic tree inferred from the four-gene dataset (*atp*6, *cox*3, *rpb*2, and *tef*1) (introns excluded), showing the position of the new genus *Rostrupomyces* in Xerocomoideae. Bootstrap support values (BS ≥ 70%) and the corresponding Bayesian posterior probabilities (PP ≥ 0.90) are shown above the supported branches. The two *Buchwaldoboletus* and seven *Chalciporus* species (subfamily Chalciporoideae) were used as outgroup. All taxa belonging to subfamilies Austroboletoideae, Boletoideae, Chalciporoideae, Leccinoideae, and Zangioideae were collapsed into subfamily clades. All generic clades in subfamily Xerocomoideae (excluding *Hemileccinum* and *Rostrupomyces*) and *Pulveroboletus* group with high supports, were also collapsed.

A total of 39 sequences were newly generated in this study and deposited in GenBank. The ML phylograms from the mitochondrial and nuclear datasets were similar in topology without any supported conflict. The Boletaceae-wide, two-genome alignment contained 743 sequences comprising four genes (146 for *atp*6, 110 for *cox*3, 231 for *rpb*2, 256 for *tef*1) from 262 voucher specimens (Table 1) corresponding to 254 species, and was 2946 characters long (DOI: 10.6084/m9.figshare.23301077). ML and BI trees of the concatenated four-character set showed similar topologies without any supported conflicts (Bootstrap Support values, BS ≥ 70% and posterior probabilities, PP ≥ 0.90; Fig. 1). In the four-gene ML phylogram, the six subfamily clades were retrieved, namely the Austroboletoideae G. Wu & Zhu L. Yang, Boletoideae Singer, Chalciporoideae G. Wu & Zhu L. Yang, Leccinoideae G. Wu & Zhu L. Yang, Xerocomoideae, and Zangioideae G. Wu, Yan C. Li & Zhu L. Yang. The *Pulveroboletus* group introduced by Wu et al. (2014, 2016) was not monophyletic; however, the monophyly of each genus in this group was highly supported. All the Xerocomus (Rostrupomyces) sisongkhramensis collections included formed a highly supported (BS = 100%, PP = 1) monophyletic group, sister to *Rubinosporus* (BS = 99%, PP = 1) clustered in subfamily Xerocomoideae with high support (BS = 99%, PP = 1). The other selected *Xerocomus* species, including the type species *X.subtomentosus* (voucher VDKO 0987), formed another, distinct monophyletic group (BS = 89%, PP = 1), sister to *Phylloporus* (BS = 79%, PP = 1). The two genera also clustered in a supported clade together with *Hourangia* (BS = 100%, PP = 1). Regarding *Hemileccinum*, all selected species formed a highly supported clade (BS = 100%, PP = 1) consisting of fourteen species-level clades, including twelve known species, one new species from Thailand (this study), and one undescribed species from China. The new species *Hemileccinuminferius* clustered in a supported clade (BS = 76%, PP = 0.98) together with the American *H.hortonii*, the Chinese *H.rugosum*, and an undescribed *Hemileccinum* species from China (voucher HKAS53421).

**Table 1.** List of collections used for DNA analyses, with origin, GenBank accession numbers, and reference(s).

Species	Voucher	Origin	*atp*6	*cox*3	*rpb*2	*tef*1	Reference(s)
Afroboletusaff.multijugus	JD671	Burundi	MH614651	MH614794	MH614747	MH614700	Vadthanarat et al. (2019b)
*Afroboletuscostatisporus*	ADK4644	Togo	KT823958	MH614795*	KT823991	KT824024	Raspé et al. (2016); Vadthanarat et al. (2019b)*
*Afroboletusluteolus*	ADK4844	Togo	MH614652	MH614796	MH614748	MH614701	Vadthanarat et al. (2019b)
*Amoenoboletusgranulopunctatus*	HKAS 86007	China	–	–	MW560079	MZ741478	Wu et al. (2021)
*Amoenoboletusgranulopunctatus*	HKAS 80250	China	–	–	MW560080	MW566746	Wu et al. (2021)
*Amylotramabanrockensis*	AD-C58672	Australia	–	–	–	MN413637	Lebel et al. (2022)
*Amylotramaclelandii*	MEL2432546	Australia	–	–	–	MN413630	Lebel et al. (2022)
*Anthracoporuscystidiatus*	HKAS55375	China	–	–	MT110410	KT990816*	Li and Yang (2021); Wu et al. (2016)*
*Anthracoporusholophaeus*	HKAS59407	China	–	–	KT990506	KT990888	Wu et al. (2016)
*Anthracoporusnigropurpureus*	HKAS52685	China	–	–	KT990459	KT990821	Wu et al. (2016)
*Aureoboletusauriflammeus*	CFMR:BOS-699	USA	–	–	MK766269	MK721060	Kuo and Ortiz-Santana (2020)
*Aureoboletuscatenarius*	HKAS54467	China	–	–	KT990349	KT990711	Wu et al. (2016)
*Aureoboletusduplicatoporus*	HKAS50498	China	–	–	KF112754	KF112230	Wu et al. (2014)
*Aureoboletusformosus*	GDGM44441	China	–	–	KT291751	KT291744	Zhang et al. (2015)
*Aureoboletusgentilis*	ADK4865	Belgium	KT823961	MH614797*	KT823994	KT824027	Raspé et al. (2016); Vadthanarat et al. (2019b)*
*Aureoboletusglutinosus*	GDGM44477	China	–	–	MH700229	MH700205	Zhang et al. (2019)
*Aureoboletusinnixus*	CFMR:BOS-544	USA	–	–	MK766270	MK721061	Kuo and Ortiz-Santana (2020)
*Aureoboletusmoravicus*	VDKO1120	Belgium	MG212528	MH614798*	MG212615	MG212573	Vadthanarat et al. (2018); Vadthanarat et al. (2019b)*
*Aureoboletusnephrosporus*	HKAS74929	China	–	–	KT990358	KT990721	Wu et al. (2016)
*Aureoboletuspseudoauriporus*	JAB 80	USA	–	–	MW737471	MW737490	Farid et al. (2021)
*Aureoboletusraphanaceus*	GDGM 53127	China	–	–	MN549706	MN549676	Zhang et al. (2019)
*Aureoboletussingeri*	CFMR:BOS-468	Belize	–	–	MK766274	MK721065	Kuo and Ortiz-Santana (2020)
*Aureoboletustenuis*	GDGM42601	China	–	–	KT291754	KT291745	Zhang et al. (2015)
*Aureoboletusthibetanus*	AFTOL-ID-450	China	DQ534600*	–	DQ366279	DQ029199	Binder and Hibbett (2006)*; Unpublished
*Aureoboletustomentosus*	HKAS90216	China	–	–	KT990355	KT990717	Wu et al. (2016)
*Aureoboletusviscidipes*	HKAS77103	China	–	–	KT990360	KT990723	Wu et al. (2016)
*Aureoboletusviscosus*	OR0361	Thailand	MH614655	MH614801	MH614751	MH614704	Vadthanarat et al. (2019b)
*Australopiluspalumanus*	REH-9433	Australia	–	–	MK766276	MK721067	Kuo and Ortiz-Santana (2020)
Austroboletuscf.dictyotus	OR0045	Thailand	KT823966	MH614802*	KT823999	KT824032	Raspé et al. (2016); Vadthanarat et al. (2019b)*
Austroboletuscf.subvirens	OR0573	Thailand	MH614656	MH614803	MH614752	MH614705	Vadthanarat et al. (2019b)
*Austroboletusolivaceoglutinosus*	HKAS57756	China	–	–	KF112764	KF112212	Wu et al. (2014)
*Baorangiamajor*	OR0209	Thailand	MG897421	MK372295*	MG897441	MG897431	Phookamsak et al. (2019); Vadthanarat et al. (2019b)*
*Baorangiapseudocalopus*	HKAS63607	China	–	–	KF112677	KF112167	Wu et al. (2014)
*Baorangiarufomaculata*	BOTH4144	USA	MG897415	MH614805*	MG897435	MG897425	Phookamsak et al. 2019; Vadthanarat et al. (2019b)*
*Binderoboletussegoi*	TWH8035	Guyana	**OP358290**	**OP358307**	–	–	**This study**
Boletellusaff.ananas	NY815459	Costa Rica	–	–	KF112760	KF112308	Wu et al. (2014)
Boletellusaff.emodensis	OR0061	Thailand	KT823970	MH614806*	KT824003	KT824036	Raspé et al. (2016); Vadthanarat et al. (2019b)*
*Boletellusananas*	K(M)123769	Belize	MH614658	MH614807	MH614754	MH614707	Vadthanarat et al. (2019b)
*Boletellusareolatus*	TNS-F-61444 or BLT-7	Japan	–	AB989025	AB999754	–	Sata and Hattori (2015)
*Boletellusaurocontextus*	TNS-F-61501 or BLT-65	Japan	–	AB989037	AB999770	–	Sata and Hattori (2015)
*Boletellusemodensis*	TNS-F-61564 or BLT-128	Japan	–	AB989053	AB999782	–	Sata and Hattori (2015)
*Boletusaereus*	VDKO1055	Belgium	MG212530	MH614809*	MG212617	MG212575	Vadthanarat et al. (2018); Vadthanarat et al. (2019b)*
*Boletusalbobrunnescens*	OR0131	Thailand	KT823973	MH614810*	KT824006	KT824039	Raspé et al. (2016); Vadthanarat et al. (2019b)*
*Boletusbotryoides*	HKAS53403	China	–	–	KT990375	KT990738	Wu et al. (2016)
*Boletusedulis*	VDKO0869	Belgium	MG212531	MH614811*	MG212618	MG212576	Vadthanarat et al. (2018); Vadthanarat et al. (2019b)*
*Boletusrubriceps*	MICH:KUO-08150719	USA	–	–	MK766284	MK721076	Kuo and Ortiz-Santana (2020)
*Borofutusdhakanus*	OR0345	Thailand	MH614660	MH614814	MH614755	MH614709	Vadthanarat et al. (2019b)
*Buchwaldoboletuslignicola*	HKAS76674	China	–	–	KF112819	KF112277	Wu et al. (2014)
*Buchwaldoboletuslignicola*	VDKO1140	Belgium	MH614661	MH614815	MH614756	MH614710	Vadthanarat et al. (2019b)
*Butyriboletusappendiculatus*	VDKO0193b	Belgium	MG212537	MH614816*	MG212624	MG212582	Vadthanarat et al. (2018); Vadthanarat et al. (2019b)*
Butyriboletuscf.roseoflavus	OR0230	China	KT823974	MH614819*	KT824007	KT824040	Raspé et al. (2016); Vadthanarat et al. (2019b)*
*Butyriboletuspseudoregius*	VDKO0925	Belgium	MG212538	MH614817*	MG212625	MG212583	Vadthanarat et al. (2018); Vadthanarat et al. (2019b)*
*Butyriboletusroseopurpureus*	BOTH4497	USA	MG897418	MH614818*	MG897438	MG897428	Phookamsak et al. (2019); Vadthanarat et al. (2019b)*
*Butyriboletussubsplendidus*	HKAS50444	China	–	–	KT990379	KT990742	Wu et al. (2016)
*Butyriboletusyicibus*	HKAS55413	China	–	–	KF112674	KF112157	Wu et al. (2014)
*Cacaoporuspallidicarneus*	SV0221	Thailand	MK372262	MK372299	MK372286	MK372273	Vadthanarat et al. (2019b)
*Cacaoporustenebrosus*	SV0223	Thailand	MK372266	MK372303	MK372290	MK372277	Vadthanarat et al. (2019b)
*Caloboletuscalopus*	ADK4087	Belgium	MG212539	MH614820	KP055030	KJ184566	Vadthanarat et al. (2018); Zhao et al. (2014a); Zhao et al. (2014b); Vadthanarat et al. (2019b)
*Caloboletusfirmus*	BOS-372	Belize	–	–	MK766288	MK721080	Kuo and Ortiz-Santana (2020)
*Caloboletusinedulis*	BOTH3963	USA	MG897414	MH614821*	MG897434	MG897424	Phookamsak et al. (2019); Vadthanarat et al. (2019b)*
*Caloboletusradicans*	VDKO1187	Belgium	MG212540	MH614822*	MG212626	MG212584	Vadthanarat et al. (2018); Vadthanarat et al. (2019b)*
*Caloboletusyunnanensis*	HKAS69214	China	–	–	KT990396	KJ184568	Zhao et al. (2014a); Wu et al. (2016)
Chalciporusaff.piperatus	OR0586	Thailand	KT823976	MH614824*	KT824009	KT824042	Raspé et al. (2016); Vadthanarat et al. (2019b)*
Chalciporusaff.rubinus	OR0139	China	MH614663	–	MH614758	MH614712	Vadthanarat et al. (2019b)
*Chalciporusafricanus*	JD517	Cameroon	KT823963	MH614825*	KT823996	KT824029	Raspé et al. (2016); Vadthanarat et al. (2019b)*
*Chalciporuspiperatus*	VDKO1063	Belgium	MH614664	MH614826	MH614759	MH614713	Vadthanarat et al. (2019b)
*Chalciporusrubinus*	AF2835	Belgium	KT823962	–	KT823995	KT824028	Raspé et al. (2016)
*Chalciporus* sp.	OR0363	Thailand	MH645586	MH645607	MH645602	MH645594	Vadthanarat et al. (2019b)
*Chalciporus* sp.	OR0373	Thailand	MH645587	MH645608	MH645603	MH645595	Vadthanarat et al. (2019b)
*Chamonixiabrevicolumna*	DBG_F28707	USA	–	–	MK766291	MK721083	Kuo and Ortiz-Santana (2020)
*Chamonixiacaespitosa*	OSC117571	USA	–	–	MK766293	MK721085	Kuo and Ortiz-Santana (2020)
*Chiuavirens*	OR0266	China	MG212541	MH614828*	MG212627	MG212585	Vadthanarat et al. (2018); Vadthanarat et al. (2019b)*
*Chiuaviridula*	HKAS74928	China	–	–	KF112794	KF112273	Wu et al. (2014)
Crocinoboletuscf.laetissimus	OR0576	Thailand	KT823975	MH614833*	KT824008	KT824041	Raspé et al. (2016); Vadthanarat et al. (2019b)*
*Crocinoboletusrufoaureus*	HKAS53424	China	–	–	KF112710	KF112206	Wu et al. (2014)
*Cupreoboletuspoikilochromus*	GS10070	Italy	–	–	KT157068	KT157072	Gelardi et al. (2015)
*Cyanoboletusbrunneoruber*	OR0233	China	MG212542	MH614834*	MG212628	MG212586	Vadthanarat et al. (2018); Vadthanarat et al. (2019b)*
*Cyanoboletuspulverulentus*	RW109	Belgium	KT823980	MH614835*	KT824013	KT824046	Raspé et al. (2016); Vadthanarat et al. (2019b)*
*Cyanoboletussinopulverulentus*	HKAS59609	China	–	–	KF112700	KF112193	Wu et al. (2014)
*Erythrophylloporusaurantiacus*	REH7271	Costa Rica	MH614666	MH614829	MH614761	MH614715	Vadthanarat et al. (2019a)
*Erythrophylloporusfagicola*	Garay215	Mexico	MH614667	MH614830	MH614762	MH614716	Vadthanarat et al. (2019a)
*Erythrophylloporuspaucicarpus*	OR1151	Thailand	MH614670	MH614831	MH614765	MH614719	Vadthanarat et al. (2019a)
*Erythrophylloporussuthepensis*	SV0236	Thailand	MH614672	MH614832	MH614767	MH614721	Vadthanarat et al. (2019a)
*Fistulinellaprunicolor*	REH9880	Australia	MH614676	MH614840	MH614771	MH614725	Vadthanarat et al. (2019b)
*Harryachromapes*	HKAS50527	China	–	–	KF112792	KF112270	Wu et al. (2014)
*Harryamoniliformis*	HKAS49627	China	–	–	KT990500	KT990881	Wu et al. (2016)
*Heimioporusconicus*	HKAS53451	China	–	–	KF112805	KF112226	Wu et al. (2016)
*Heimioporusaustralis*	REH9288	Australia	–	–	–	KP327703	Halling et al. (2015)
*Heimioporuscooloolae*	REH9817	Australia	–	–	–	KP327710	Halling et al. (2015)
*Heimioporusfruticicola*	REH8962	Australia	–	–	–	KP327696	Halling et al. (2015)
*Heimioporusgaojiaocong*	HKAS80582	China	–	–	KT990409	KT990770	Wu et al. (2016)
*Heimioporusivoryi*	REH8620	Costa Rica	–	–	–	KP327683	Halling et al. (2015)
*Heimioporusjaponicus*	OR0114	Thailand	KT823971	–	KT824004	KT824037	Raspé et al. (2016)
*Heimioporusjaponicus*	SV0016	Thailand	MT136776	–	MT136766	MT136771	Vadthanarat et al. (2020)
*Heimioporusmandarinus*	OR0218	Thailand	MG212546	–	MG212632	MG212590	Vadthanarat et al. (2018)
*Heimioporussubcostatus*	SV0235	Thailand	MT136780	–	MT136770	MT136775	Vadthanarat et al. (2020)
*Hemileccinumalbidum*	KUN-HKAS81120	China	–	–	MZ936320	MZ936352	Li et. al. (2021)
*Hemileccinuminferius*	BR0260	Thailand	**OP358291**	–	**OP358312**	**OP358319**	**This study**
*Hemileccinuminferius*	SV0282	Thailand	**OP358292**	–	–	–	**This study**
*Hemileccinumbrevisporum*	KUN-HKAS89150	China	–	–	MZ936328	MZ936362	Li et. al. (2021)
*Hemileccinumbrevisporum*	HKAS59445	China	–	–	KT990414	KT990775	Wu et al. (2016)
*Hemileccinumdepilatum*	AF2845	Belgium	MG212547	MH614843*	MG212633	MG212591	Vadthanarat et al. (2018); Vadthanarat et al. (2019b)*
*Hemileccinumferrugineipes*	KUN-HKAS115554	China	–	–	MZ936330	MZ973011	Li et. al. (2021)
*Hemileccinumfloridanum*	AB16	USA	–	–	–	MW737481	Farid et al. (2021)
*Hemileccinumhortonii*	MICH:KUO-07050706	USA	–	–	MK766377	MK721175	Kuo and Ortiz-Santana (2020)
*Hemileccinumimpolitum*	ADK4078	Belgium	MG212548	MH614844*	MG212634	MG212592	Vadthanarat et al. (2018); Vadthanarat et al. (2019b)*
*Hemileccinumindecorum*	OR0863	Thailand	MH614677	MH614845	MH614772	MH614726	Vadthanarat et al. (2019b)
*Hemileccinumparvum*	KUN-HKAS115553	China	–	–	MZ936333	MZ973010	Li et. al. (2021)
*Hemileccinumrubropunctum*	REH-8501	USA	–	–	MK766327	MK721122	Kuo and Ortiz-Santana (2020)
*Hemileccinumrugosum*	HKAS84355	China	–	–	KT990413	KT990774	Wu et al. (2016)
*Hemileccinum* sp.	HKAS53421	China	–	–	KF112751	KF112235	Wu et al. (2014)
*Hemileccinumsubglabripes*	MICH:KUO-07230802	USA	–	–	MK766300	MK721092	Kuo and Ortiz-Santana (2020)
*Hortiboletusamygdalinus*	HKAS54166	China	–	–	KT990416	KT990777	Wu et al. (2016)
*Hortiboletuscampestris*	MICH:KUO-08240502	USA	–	–	MK766302	MK721094	Kuo and Ortiz-Santana (2020)
*Hortiboletusrubellus*	VDKO0403	Belgium	MH614679	MH614847	MH614774	–	Vadthanarat et al. (2019b)
*Hortiboletussubpaludosus*	HKAS59608	China	–	–	KF112696	KF112185	Wu et al. (2014)
Hourangiacf.pumila	OR0762	Thailand	MH614680	MH614848	MH614775	MH614728	Vadthanarat et al. (2019b)
*Hourangiacheoi*	HKAS52269	China	–	–	KF112773	KF112286	Zhu et al. (2015)
*Hourangiamicrocarpa*	HKAS53378	China	–	–	KF112775	KF112300	Wu et al. (2014)
*Hourangianigropunctata*	HKAS 57427	China	–	–	KP136978	KP136927	Zhu et al. (2015)
*Hymenoboletusluteopurpureus*	HKAS46334	China	–	–	KF112795	KF112271	Wu et al. (2014)
*Imleriabadia*	VDKO0709	Belgium	KT823983	MH614849*	KT824016	KT824049	Raspé et al. (2016); Vadthanarat et al. (2019b)*
*Imleriaobscurebrunnea*	OR0263	China	MH614681	MH614850	MH614776	MH614729	Vadthanarat et al. (2019b)
*Imleriapallidus*	BOTH4356	USA	MH614659	MH614812	–	MH614708	Vadthanarat et al. (2019b)
*Indoporussquamulosus*	HKAS107153	China	–	–	MT110409	MT110335	Li and Yang (2021)
*Ionosporuslongipes*	LEE1180	Malaysia	MT085461	–	MH712031*	MT085471	Chuankid et al. (2021); Khmelnitsky et al. (2019)
*Kaziboletusrufescens*	HKAS74706	Bangladesh	–	–	JQ928600	JQ928578	Hosen et al. (2021)
*Lanmaoaangustispora*	HKAS74752	China	–	–	KM605177	KM605154	Wu et al. (2015)
*Lanmaoaasiatica*	OR0228	China	MH614682	MH614851	MH614777	MH614730	Vadthanarat et al. (2019b)
*Lanmaoacarminipes*	BOTH4591	USA	MG897419	MH614852*	MG897439	MG897429	Phookamsak et al. (2019); Vadthanarat et al. (2019b)*
*Lanmaoapallidorosea*	BOTH4432	USA	MG897417	MH614853*	MG897437	MG897427	Phookamsak et al. (2019); Vadthanarat et al. (2019b)*
*Lanmaoasublurida*	Farid 1023	USA	–	–	MW737460	MW737485	Farid et al. (2021)
Leccinellumaff.crocipodium	HKAS76658	China	–	–	KF112728	KF112252	Wu et al. (2014)
Leccinellumaff.griseum	KPM-NC-0017832	Japan	KC552164	–	–	JN378450*	Unpublished; Orihara et al. (2012)*
*Leccinellumcremeum*	HKAS90639	China	–	–	KT990420	KT990781	Wu et al. (2016)
*Leccinumscabrum*	VDKO0938	Belgium	MG212549	MH614858*	MG212635	MG212593	Vadthanarat et al. (2018); Vadthanarat et al. (2019b)*
*Leccinumschistophilum*	VDKO1128	Belgium	KT823989	MH614859*	KT824022	KT824055	Raspé et al. (2016); Vadthanarat et al. (2019b)*
*Leccinumvariicolor*	VDKO0844	Belgium	MG212550	MH614860*	MG212636	MG212594	Vadthanarat et al. (2018); Vadthanarat et al. (2019b)*
*Leccinumversipelle*	KPM-NC-0017833	Scotland	KC552172	–	–	JN378454	Orihara et al. (2016); Orihara et al. (2012)
*Leccinumvulpinum*	KPM-NC-0017834	Scotland	KC552171	–	–	JN378456	Orihara et al. (2016); Orihara et al. (2012)
*Mucilopiluscastaneiceps*	HKAS75045	China	–	–	KF112735	KF112211	Wu et al. (2014)
*Mucilopilusparacastaneiceps*	HKAS50338	China	–	–	KT990391	KT990755	Wu et al. (2016)
*Mucilopilusruber*	HKAS84555	China	–	–	MT110436	MT110364	Li and Yang (2021)
*Mycoamaranthuscambodgensis*	SV0197	Thailand	MZ355900	MZ355909	–	–	Vadthanarat et al. (2022)
*Neoboletusbrunneissimus*	OR0249	China	MG212551	MH614861*	MG212637	MG212595	Vadthanarat et al. (2018); Vadthanarat et al. (2019b)*
*Neoboletusferrugineus*	HKAS77718	China	–	–	KT990431	KT990789	Wu et al. (2016)
*Neoboletusflavidus*	HKAS59443	China	–	–	KU974144	KU974136	Wu et al. (2016)
*Neoboletushainanensis*	HKAS59469	China	–	–	KF112669	KF112175	Wu et al. (2014)
*Neoboletusjunquilleus*	AF2922	France	MG212552	MH614862*	MG212638	MG212596	Vadthanarat et al. (2018); Vadthanarat et al. (2019b)*
*Neoboletusmagnificus*	HKAS74939	China	–	–	KF112653	KF112148	Wu et al. (2014)
*Neoboletusobscureumbrinus*	OR0553	Thailand	MK372271	–	MK372294	MK372282	Vadthanarat et al. (2019b)
*Neoboletustomentulosus*	HKAS53369	China	–	–	KF112659	KF112154	Wu et al. (2014)
*Neoboletuserythropus*	VDKO0690	Belgium	KT823982	MH614864*	KT824015	KT824048	Raspé et al. (2016); Vadthanarat et al. (2019b)*
*Octavianiaasterosperma*	AQUI3899	Italy	KC552159	–	–	KC552093	Orihara et al. (2016)
*Octavianiacyanescens*	PNW-FUNGI-5603	USA	KC552160	–	–	JN378438	Orihara et al. (2016); Orihara et al. (2012)
*Octavianiatasmanica*	MEL2128484	Australia	KC552157	–	–	JN378437	Orihara et al. (2016); Orihara et al. (2012)
*Octavianiazelleri*	MES270	USA	KC552161	–	–	JN378440	Orihara et al. (2016); Orihara et al. (2012)
*Parvixerocomuspseudoaokii*	OR0155	China	MG212553	MH614865	MG212597	MG212597	Vadthanarat et al. (2019b)
*Paxilloboletuslatisporus*	ADK5072	Congo	–	–	MZ707870	MZ707866	Badou et al. (2022)
*Paxilloboletusafricanus*	SAB0716	Guinea	–	–	MZ707869	MZ707865	Badou et al. (2022)
*Phylloporusbellus*	OR0473	China	MH580778	MH614866*	MH580818	MH580798	Chuankid et al. (2019); Vadthanarat et al. (2019b)*
*Phylloporusbrunneiceps*	OR0050	Thailand	KT823968	MH614867*	KT824001	KT824034	Raspé et al. (2016); Vadthanarat et al. (2019b)*
*Phylloporuscastanopsidis*	OR0052	Thailand	KT823969	MH614868*	KT824002	KT824035	Raspé et al. (2016); Vadthanarat et al. (2019b)*
*Phylloporusmaculatus*	OR0285	China	MH580780	–	MH580820	MH580800	Chuankid et al. (2019)
*Phylloporuspachycystidiatus*	HKAS53422	China	–	–	KF112777	KF112288	Wu et al. (2014)
*Phylloporuspelletieri*	WU18746	Austria	MH580781	MH614869*	MH580821	MH580801	Chuankid et al. (2019); Vadthanarat et al. (2019b)*
*Phylloporuspusillus*	OR1158	Thailand	MH580783	MH614870*	MH580823	MH580803	Chuankid et al. (2019); Vadthanarat et al. (2019b)*
*Phylloporusrhodoxanthus*	WU17978	Austria	MH580785	MH614871*	MH580824	MH580805	Chuankid et al. (2019); Vadthanarat et al. (2019b)*
*Phylloporusrubeolus*	OR0251	China	MH580786	MH614872*	MH580825	MH580806	Chuankid et al. (2019); Vadthanarat et al. (2019b)*
*Phylloporusrubiginosus*	OR0169	China	MH580788	MH614873*	MH580827	MH580808	Chuankid et al. (2019); Vadthanarat et al. (2019b)*
*Phylloporusrubrosquamosus*	HKAS52552	China	–	–	KF112780	KF112289	Wu et al. (2014)
*Phylloporusscabripes*	CFMR:BOS-621	Belize	–	–	MK766359	MK721156	Kuo and Ortiz-Santana (2020)
*Phylloporussubbacillisporus*	OR0436	China	MH580792	MH614875*	MH580831	MH580812	Chuankid et al. (2019); Vadthanarat et al. (2019b)*
*Phylloporussubrubeolus*	BC022	Thailand	MH580793	MH614876*	MH580832	MH580813	Chuankid et al. (2019); Vadthanarat et al. (2019b)*
*Phylloporusyunnanensis*	OR0448	China	MG212554	MH614877*	MG212640	MG212598	Vadthanarat et al. (2018); Vadthanarat et al. (2019b)*
*Porphyrelluscastaneus*	OR0241	China	MG212555	MH614878*	MG212641	MG212599	Vadthanarat et al. (2018); Vadthanarat et al. (2019b)*
*Porphyrellusporphyrosporus*	MB97 023	Germany	DQ534609	–	GU187800	GU187734	Binder and Hibbett (2006); Binder et al. (2010)
*Pseudoaustroboletusvalens*	HKAS82644	China	–	–	MT110431	MT110359	Li and Yang (2021)
*Pulchroboletussclerotiorum*	FLAS F 60333	USA	–	–	MF614169	MF614167	Crous et al. (2019)
*Pulchroboletussclerotiorum*	FLAS F 60334	USA	–	–	MF614164	MF614165	Crous et al. (2019)
Pulveroboletusaff.ravenelii	ADK4360	Togo	KT823957	MH614882*	KT823990	KT824023	Raspé et al. (2016); Vadthanarat et al. (2019b)*
Pulveroboletusaff.ravenelii	ADK4650	Togo	KT823959	MH614883*	KT823992	KT824025	Raspé et al. (2016); Vadthanarat et al. (2019b)*
*Pulveroboletusbrunneopunctatus*	HKAS55369	China	–	–	KT990455	KT990814	Wu et al. (2016)
*Pulveroboletusfragrans*	OR0673	Thailand	KT823977	MH614884*	KT824010	KT824043	Raspé et al. (2016); Vadthanarat et al. (2019b)*
*Pulveroboletusravenelii*	REH2565	USA	KU665635	MH614885*	KU665637	KU665636	Raspé et al. (2016); Vadthanarat et al. (2019b)*
Retiboletusaff.nigerrimus	OR0049	Thailand	KT823967	MH614886*	KT824000	KT824033	Raspé et al. (2016); Vadthanarat et al. (2019b)*
*Retiboletusbrevibasidiatus*	OR0570	Thailand	MT085469	–	MT085479	MT085476	Chuankid et al. (2021)
*Retiboletusbrunneolus*	HKAS52680	China	–	–	KF112690	KF112179	Wu et al. (2014)
*Retiboletusfuscus*	OR0231	China	MG212556	MH614887*	MG212642	MG212600	Vadthanarat et al. (2018); Vadthanarat et al. (2019b)*
*Retiboletusgriseus*	MB03 079	USA	KT823964	MH614888*	KT823997	KT824030	Raspé et al. (2016); Vadthanarat et al. (2019b)*
*Retiboletuskauffmanii*	OR0278	China	MG212557	MH614889*	MG212643	MG212601	Vadthanarat et al. (2018); Vadthanarat et al. (2019b)*
*Retiboletusnigerrimus*	HKAS53418	China	–	–	KT990462	KT990824	Wu et al. (2016)
*Rhodactinahimalayensis*	CMU25117	Thailand	MG212558	–	–	MG212602, MG212603	Vadthanarat et al. (2018)
*Rhodactinarostratispora*	SV0170	Thailand	MG212560	–	MG212645	MG212605	Vadthanarat et al. (2018)
*Rossbeeveracryptocyanea*	KPM-NC17843	Japan	KT581441	–	–	KC552072	Orihara et al. (2016)
*Rossbeeveraeucyanea*	TNS-F-36986	Japan	KC552115	–	–	KC552068	Orihara et al. (2016)
*Rossbeeveragriseovelutina*	TNS-F-36989	Japan	KC552124	–	–	KC552076	Orihara et al. (2016)
*Rossbeeverapachydermis*	KPM-NC23336	New Zealand	KJ001064	–	–	KP222912	Orihara et al. (2016)
*Rossbeeveravittatispora*	TO-AUS-72	Australia	KC552108	–	–	KC552065	Orihara et al. (2016)
*Rostrupomycessisongkhramensis*	BR0311	Thailand	**OP358293**	–	**OP358313**	**OP358320**	**This study**
*Rostrupomycessisongkhramensis*	BR0313	Thailand	**OP358294**	–	**OP358314**	**OP358321**	**This study**
*Rostrupomycessisongkhramensis*	BR0368	Thailand	**OP358295**	–	–	–	**This study**
*Rostrupomycessisongkhramensis*	BR0371	Thailand	**OP358296**	–	–	**OP358322**	**This study**
*Rostrupomycessisongkhramensis*	OR0915	Thailand	**OP358297**	–	–	–	**This study**
*Rostrupomycessisongkhramensis*	OR0918	Thailand	**OP358298**	–	–	–	**This study**
*Rostrupomycessisongkhramensis*	OR0919	Thailand	**OP358299**	**OP358308**	**OP358315**	**OP358323**	**This study**
*Rostrupomycessisongkhramensis*	OR1004	Thailand	**OP358300**	–	–	–	**This study**
*Rostrupomycessisongkhramensis*	OR1059	Thailand	**OP358301**	–	–	–	**This study**
*Rostrupomycessisongkhramensis*	OR1392	Thailand	**OP358302**	–	–	–	**This study**
*Rostrupomycessisongkhramensis*	OR1399	Thailand	**OP358303**	–	–	–	**This study**
*Rostrupomycessisongkhramensis*	SV0155	Thailand	**OP358304**	**OP358309**	**OP358316**	**OP358324**	**This study**
*Rostrupomycessisongkhramensis*	SV0219	Thailand	**OP358305**	**OP358310**	**OP358317**	**OP358325**	**This study**
*Rostrupomycessisongkhramensis*	SV0225	Thailand	**OP358306**	**OP358311**	**OP358318**	**OP358326**	**This study**
*Royoungiarubina*	HKAS53379	China	–	–	KF112796	KF112274	Wu et al. (2014)
*Rubinosporusauriporus*	SV0101	Thailand	MZ355897	MZ355906	MZ355904	MZ355902	Vadthanarat et al. (2022)
*Rubinosporusauriporus*	SV0090	Thailand	MZ355896	MZ355905	MZ355903	MZ355901	Vadthanarat et al. (2022)
*Rubroboletuslegaliae*	VDKO0936	Belgium	KT823985	MH614890*	KT824018	KT824051	Raspé et al. (2016); Vadthanarat et al. (2019b)*
*Rubroboletusrhodosanguineus*	BOTH4263	USA	MG897416	MH614891*	MG897436	MG897426	Phookamsak et al. (2019); Vadthanarat et al. (2019b)*
*Rubroboletusrhodoxanthus*	HKAS84879	China	–	–	KT990468	KT990831	Wu et al. (2016)
*Rubroboletussatanas*	VDKO0968	Belgium	KT823986	MH614892*	KT824019	KT824052	Raspé et al. (2016); Vadthanarat et al. (2019b)*
*Rugiboletusandinus*	REH-7705	Costa Rica	–	–	MK766316	MK721111	Kuo and Ortiz-Santana (2020)
*Rugiboletusbrunneiporus*	HKAS83209	China	–	–	KM605168	KM605144	Wu et al. (2015)
*Rugiboletusextremiorientalis*	OR0406	Thailand	MG212562	MH614893*	MG212647	MG212607	Vadthanarat et al. (2018); Vadthanarat et al. (2019b)*
*Singerocomusinundabilis*	TWH9199	Guyana	MH645588	MH645609	LC043089*	MH645596	Henkel et al. (2016)*; Vadthanarat et al. (2019b)
*Singerocomusrubriflavus*	TWH9585	Guyana	MH645589	MH645610	–	MH645597	Vadthanarat et al. (2019b)
*Spongiformathailandica*	DED7873	Thailand	MG212563	MH614894**	MG212648	KF030436*	Nuhn et al. (2013)*; Vadthanarat et al. (2018); Vadthanarat et al. (2019b)**
*Spongisporatemasekensis*	SING 0206334	Singapore	–	–	MG674378	MG674377	Wu et al. (2018)
*Spongisporatemasekensis*	ACMF5	Singapore	MZ803018	–	MZ824748	MZ803023	Raghoonundon et al. (2021)
*Strobilomycesatrosquamosus*	HKAS55368	China	–	–	KT990476	KT990839	Wu et al. (2016)
*Strobilomycesechinocephalus*	OR0243	China	MG212564	–	MG212649	MG212608	Vadthanarat et al. (2018)
*Strobilomycesfloccopus*	RW103	Belgium	KT823978	MH614895*	KT824011	KT824044	Raspé et al. (2016); Vadthanarat et al. (2019b)*
*Strobilomycesmirandus*	OR0115	Thailand	KT823972	MH614896*	KT824005	KT824038	Raspé et al. (2016); Vadthanarat et al. (2019b)*
*Strobilomycesverruculosus*	HKAS55389	China	–	–	KF112813	KF112259	Wu et al. (2014)
*Suillellusluridus*	VDKO0241b	Belgium	KT823981	MH614901*	KT824014	KT824047	Raspé et al. (2016); Vadthanarat et al. (2019b)*
*Suillellusqueletii*	VDKO1185	Belgium	MH645590	MH645611	MH645604	MH645598	Vadthanarat et al. (2019b)
*Suillellussubamygdalinus*	HKAS57262	China	–	–	KF112660	KF112174	Wu et al. (2014)
*Sutoriusaustraliensis*	REH9441	Australia	MG212567	MK386576**	MG212652	JQ327032*	Halling et al. (2012)*; Vadthanarat et al. (2018); Vadthanarat et al. (2019b)**
*Sutoriuseximius*	REH9400	USA	MG212568	MH614902**	MG212653	JQ327029*	Halling et al. (2012)*; Vadthanarat et al. (2018); Vadthanarat et al. (2019b)**
*Sutoriuspachypus*	OR0411	Thailand	MN067465	–	MN067500	MN067484	Vadthanarat et al. (2021)
*Sutoriuspseudotylopilus*	OR0378B	Thailand	MH614692	MH614903	MH614787	MH614740	Vadthanarat et al. (2019b)
*Sutoriusrubinus*	OR0379	Thailand	MH614693	MH614904	MH614788	MH614741	Vadthanarat et al. (2019b)
*Sutoriusubonensis*	SV0032	Thailand	MN067472	–	MN067507	MN067491	Vadthanarat et al. (2021)
*Tengioboletusglutinosus*	HKAS53425	China	–	–	KF112800	KF112204	Wu et al. (2014)
*Tengioboletusreticulatus*	HKAS53426	China	–	–	KF112828	KF112313	Wu et al. (2014)
*Turmalineapersicina*	KPM-NC18001	Japan	KC552130	–	–	KC552082	Orihara et al. (2016)
*Turmalineayuwanensis*	KPM-NC18011	Japan	KC552138	–	–	KC552089	Orihara et al. (2016)
*Tylocinumgriseolum*	HKAS50281	China	–	–	KF112730	KF112284	Wu et al. (2014)
*Tylopilusatripurpureus*	HKAS50208	China	–	–	KF112799	KF112283	Wu et al. (2014)
*Tylopilusfelleus*	VDKO0992	Belgium	KT823987	MH614906*	KT824020	KT824053	Raspé et al. (2016); Vadthanarat et al. (2019b)*
*Tylopilusferrugineus*	BOTH3639	USA	MH614694	MH614907	MH614789	MH614742	Vadthanarat et al. (2019b)
*Tylopilusotsuensis*	HKAS53401	China	–	–	KF112797	KF112224	Wu et al. (2014)
*Tylopilusvinaceipallidus*	OR0137	China	MG212571	MH614912*	MG212656	MG212613	Vadthanarat et al. (2018); Vadthanarat et al. (2019b)*
*Tylopilusviolaceobrunneus*	HKAS89443	China	–	–	KT990504	KT990886	Wu et al. (2016)
*Veloboletuslimbatus*	REH9228	Australia	MT747398	–	MT747397	MN413636	Crous et al. (2019)
*Veloporphyrellusconicus*	REH8510	Belize	MH614698	MH614913	MH614792	MH614745	Vadthanarat et al. (2019b)
*Veloporphyrellusgracilioides*	HKAS53590	China	–	–	KF112734	KF112210	Wu et al. (2014)
*Veloporphyrelluspseudovelatus*	HKAS59444	China	JX984519	–	–	JX984553	Li et al. (2014)
*Veloporphyrellusvelatus*	HKAS63668	China	JX984523	–	–	JX984554	Li et al. (2014)
*Xanthoconiumaffine*	NY00815399	USA	–	–	KT990486	KT990850	Wu et al. (2016)
*Xanthoconiumpurpureum*	MICH:KUO-07061405	USA	–	–	MK766372	MK721170	Kuo and Ortiz-Santana (2020)
*Xanthoconiumsinense*	HKAS77651	China	–	–	KT990488	KT990853	Wu et al. (2016)
*Xerocomelluschrysenteron*	VDKO0821	Belgium	KT823984	MH614914*	KT824017	KT824050	Raspé et al. (2016); Vadthanarat et al. (2019b)*
*Xerocomelluscisalpinus*	ADK4864	Belgium	KT823960	MH614915*	KT823993	KT824026	Raspé et al. (2016); Vadthanarat et al. (2019b)*
*Xerocomelluscommunis*	HKAS50467	China	–	–	KT990494	KT990858	Wu et al. (2016)
*Xerocomellusripariellus*	VDKO0404	Belgium	MH614699	MH614916	MH614793	MH614746	Vadthanarat et al. (2019b)
*Xerocomusferrugineus*	CFMR:BOS-545	USA	–	–	MK766375	MK721173	Kuo and Ortiz-Santana (2020)
*Xerocomusfulvipes*	HKAS76666	China	–	–	KF112789	KF112292	Wu et al. (2014)
*Xerocomusmagniporus*	HKAS58000	China	–	–	KF112781	KF112293	Wu et al. (2014)
*Xerocomusrugosellus*	HKAS58865	China	–	–	KF112784	KF112294	Wu et al. (2014)
Xerocomusspadiceusvar.gracilis	MICH:KUO-07080702	USA	–	–	MK766378	MK721176	Kuo and Ortiz-Santana (2020)
*Xerocomussubtomentosus*	VDKO0987	Belgium	MG212572	MH614919*	MG212657	MG212614	Vadthanarat et al. (2018); Vadthanarat et al. (2019b)*
*Xerocomustenax*	MICH:KUO-08241404	USA	–	–	MK766379	MK721177	Kuo and Ortiz-Santana (2020)
*Zangiacitrina*	HKAS52684	China	HQ326850	–	–	HQ326872	Li et al. (2011)
*Zangiaolivaceobrunnea*	HKAS52272	China	HQ326857	–	–	HQ326876	Li et al. (2011)
*Zangiaroseola*	HKAS51137	China	HQ326858	–	–	HQ326877	Li et al. (2011)

**Figure 2.**
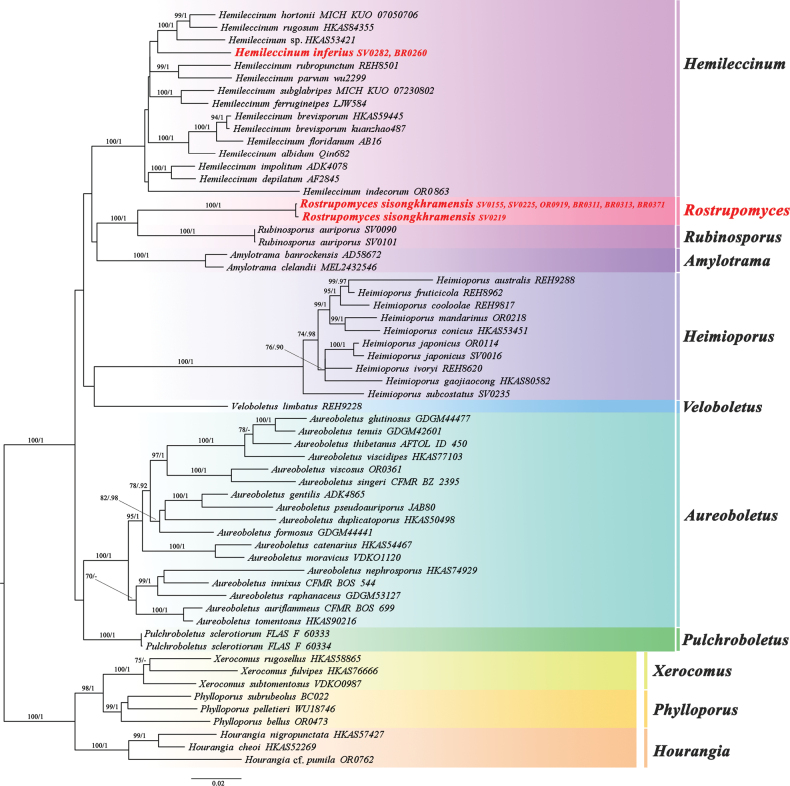
Xerocomoideae-wide phylogenetic tree inferred from the four-gene dataset (*atp*6, *cox*3, *rpb*2, and *tef*1) (introns included), including new genus *Rostrupomyces* and selected Xerocomoideae using Maximum Likelihood and Bayesian Inference methods (ML tree is presented). The three *Hourangia*, three *Phylloporus*, and three *Xerocomus* species in Xerocomoideae were used as outgroup. Bootstrap support values (BS ≥ 70%) and posterior probabilities (PP ≥ 0.90) are shown above the supported branches.

For the subfamily Xerocomoideae-wide phylogeny, no supported topological incongruence between the character sets was detected. Then, the Xerocomoideae-wide phylogeny was inferred based on the alignment containing 155 sequences of four genes (22 for *atp*6, 20 for *cox*3, 53 for *rpb*2, 60 for *tef*1) from 60 voucher specimens corresponding to 55 taxa, and was 3,161 characters long (DOI: 10.6084/m9.figshare.23301077). The ML and BI tree topologies of the concatenated five-character-set alignment were similar without any supported conflict (Fig. 2). The Xerocomoideae-wide ML tree also showed a similar topology to the Boletaceae-wide tree. However, in this subfamily Xerocomoideae-wide tree, the support of the clade consisting of the new species *Hemileccinuminferius*, *H.hortonii*, *H.rugosum*, and an undescribed *Hemileccinum* species, was lower (BS = 53%, PP = 0.71) than in the Boletaceae-wide ML tree.

### ﻿Taxonomy

#### ﻿ Rostrupomyces

Taxon classificationFungiBoletalesBoletaceae

Vadthanarat & Raspé gen. nov.

5C87B66F-8148-591B-93C2-A5F61881E434

849050

##### Etymology.

Named in honor of Frederik Georg Emil Rostrup (1831–1907), Danish botanist, mycologist, and plant pathologist, celebrating the 120 years of his describing the first new species of Boletaceae from Thailand in 1902.

##### Diagnosis.

Differs from other genera in Boletaceae by the following combination of characters: rugulose to subrugulose pileus surface, white pore when young becoming grayish yellow in age, subscabrous stipe surface with scattered granulose squamules, white basal mycelium, unchanging color in any parts, yellowish brown spore print, and broadly ellipsoid to ellipsoid, smooth basidiospores.

##### Description.

***Basidiomata*** stipitate-pileate. ***Pileus*** convex then plano-convex to plane; ***surface*** at first rugulose then subrugulose in age, finely tomentose to tomentose, dark brown to reddish brown, becoming light brown to brown to grayish orange, unchanging when bruised; ***context*** off-white then yellowish to dull pale orange in age, unchanging when cut. ***Stipe*** central, terete, cylindrical; ***surface*** subscabrous, yellowish white to pale yellow to orange white, with scattered brown to dark brown to reddish brown granulose squamules, unchanging when bruised; ***basal mycelium*** white; ***context*** solid, white becoming off-white to yellowish white in age, unchanging when cut. ***Hymenophore*** tubulate, slightly depressed to depressed around the stipe. ***Tubes*** pale yellow then grayish yellow, separable from the pileus context, unchanging when cut. ***Pores*** roundish then subangular to angular with age; when young white then yellowish white becoming grayish yellow, unchanging when touched. ***Spore print*** yellowish brown. ***Basidiospores*** ellipsoid to broadly ellipsoid, thin-walled, smooth under light microscope and SEM. ***Basidia*** 4-spored, clavate without basal clamp connection. ***Cheilo*- *and pleurocystidia*** narrowly fusiform to fusiform or narrowly utriform, thin-walled. ***Pileipellis*** an intricate trichoderm, made of moderately interwoven to loosely interwoven, thin-walled hyphae. ***Stipitipellis*** arranged parallel to the surface of the stipe, composed of moderately interwoven, thin-walled hyphae, with scattered groups of rising cells to clusters of narrowly clavate to clavate cells. ***Clamp connections*** not seen in any tissue.

##### Typus generis.

*Rostrupomycessisongkhramensis* (Khamsuntorn, Pinruan & Luangsa-ard) Vadthanarat, Raghoonundon & Raspé.

##### Distribution.

Currently known only from northern and northeastern Thailand.

##### Notes.

*Rostrupomyces* can be morphologically separated from *Xerocomus* by the different shape and surface of basidiospores, which are ellipsoid to broadly ellipsoid with smooth under light microscope and SEM in the new genus, whereas *Xerocomus* produce more or less oblong to fusiform basidiospores, usually with bacillate surface under SEM (Wu et. al. 2016). *Rostrupomyces* also produces yellowish brown spore print, whereas *Xerocomus* produces olive-brown spore print. Moreover, color change upon bruising does not occur in any part of *Rostrupomyces* basidiomes, whereas context and hymenophore of *Xerocomus* always turn more or less bluish to blue when bruised or cut (Wu et. al. 2016). The most resembling genus, *Hemileccinum*, shares some similar characters including rugulose to subrugulose pileus surface, yellow hymenophore which is depressed around the stipe apex, subscabrous stipe surface (less so in *Hemileccinum*), white basal mycelium, mostly unchanging color in any parts. However, *Rostrupomyces* can be morphologically distinguished from *Hemileccinum* by the differences in spore print color, and in the shape and surface of basidiospores. *Rostrupomyces* produces yellowish brown spore print, broadly ellipsoid to ellipsoid basidiospores with smooth surface under light microscope and SEM. *Hemileccinum* produces olive-brown spore prints, boletoid basidiospores that are smooth under light microscope, but ornamented with irregular warts and pinholes under SEM. Also, the pore surface of *Rostrupomyces* is white in young basidiomata and becomes pale yellow when mature whereas in *Hemileccinum*, the pore surface is yellow in all stages (Šutara 2008; Wu et al. 2016; Farid et al. 2021; Li et al. 2021).

#### ﻿ Rostrupomyces sisongkhramensis

Taxon classificationFungiBoletalesBoletaceae

(Khamsuntorn, Pinruan & Luangsa-ard) Vadthanarat, Raghoonundon & Raspé comb. nov.

969DD59E-D8FC-5B41-8C3D-421C9B13F68C

851393

Figs 3 , 4 , 5A–B

Xerocomus sisongkhramensis Khamsuntorn, Pinruan & Luangsa-ard. Basionym.

##### Diagnosis.

*Rostrupomycessisongkhramensis* is characterised by having dark to reddish brown, becoming brown to grayish orange pileus, with rugulose to subrugulose, finely tomentose to tomentose surface; yellowish to orange white, subscabrous, longitudinally fissurate stipe surface, with moderately scattered brown to dark brown to reddish brown granulose squamules; yellow hymenophore; unchanging color in any parts; yellowish brown spore print; and broadly ellipsoid to ellipsoid smooth basidiospores.

##### Description.

***Basidiomata*** medium-sized. ***Pileus*** 37–94(118) mm in diameter, convex at first then plano-convex to plane, sometimes with sub-depressed at the centre; ***margin*** inflexed at first then deflexed in age, exact or slightly exceeding (up to 1 mm); ***surface*** at first rugulose especially near the margin then subrugulose in age, dull, dry to moist, finely tomentose to tomentose covered with greenish yellow (3A3–4, 3B4) matted hyphae at places (especially when young), at first dark brown to reddish brown (6–8F4–8), becoming light brown to brown to grayish orange (6D/E5–6, 5B4–5) on light yellow to brownish orange (4A3–5, 5C4) background in age, gradually paler to the margin, unchanging when bruised; ***context*** (3)5–10(14) mm thick half-way to the margin, at first firm then soft in age, color distribution even, at first off-white, slightly brownish (7D/E4–5) near the pileipellis, then yellowish to orange white (4–5A2) or occasionally yellowish (3A3–4) above the hymenium especially in age, unchanging when cut. ***Stipe*** (33)41–97(108) × 6(7)–19(20) mm, central, terete, usually cylindrical for the most part but often with wider base, rarely club-shaped; ***surface*** subscabrous longitudinally fissurate, slightly shiny, yellowish white to pale yellow to orange white (3A3 to 4A2 to 5A2), occasionally pale yellow (3A3–4) near the cap, with moderately scattered brown to dark brown to reddish brown (7D/E/F4–7) granulose squamules, unchanging when bruised; ***basal mycelium*** little developed, white (1A1); ***context*** solid, firm, at first white (1A1) becoming off-white to yellowish white (4A2) occasionally pale yellow (3A3–4) especially in the above part near the stipe surface in age, yellowish to orange gray (4–5B2–3) virgate at places, unchanging when cut. ***Hymenophore*** tubulate, slightly depressed to depressed around the stipe, with slightly decurrent tooth, sometimes almost free, mostly segmentiform to subventricose. ***Tubes*** (3)4–13 mm long half-way to the margin, at first pale yellow (4A3) then grayish yellow (4B3) when mature, separable from the pileus context, unchanging when cut. ***Pores*** 0.2–0.8(1.3) mm wide half-way to the margin, irregularly arranged, roundish then subangular to angular in age; topography subregular, composite pores frequent; color distribution even, when young white (1A1) then yellowish white (4A2) becoming grayish yellow (4B3–5) infrequently with reddish brown spots (7–8E/F8) at places in age, unchanging when touched. ***Odour*** mild fungoid. ***Taste*** mild. ***Spore print*** yellowish brown (5F5) in mass.

***Macrochemical reactions***: KOH, brownish orange on pileus, yellowish to pale dull orange on pileus context and stipe surface, none or yellowish on stipe context, yellowish brown to brownish orange on hymenium; NH_4_OH, yellowish to brownish orange (occasionally with purple aura) on pileus, yellowish to pale orange on stipe surface, yellowish to brownish on hymenium, none or yellowish on pileus context and stipe context.

***Spores*** [591/10/10] (6.3–)6.9–7.9–9.1(–9.8) × (4.5–)4.8–5.5–6.2(–6.5) µm *Q* = (1.2–)1.29–1.44–1.63(–1.79). From the type (6.5–)6.9–7.7–8.8(–9.5) × (4.7–)5–5.5–6.2(–6.5) µm, *Q* = (1.2–)1.25–1.41–1.54(–1.63), *N* = 106, broadly ellipsoid to ellipsoid, thin-walled, smooth under light microscope and SEM, yellowish hyaline in water or KOH, inamyloid. ***Basidia*** 4-spored, (22–)22–26–31(–31) × (9–)9–11–13(–13) µm, clavate without basal clamp connection, hyaline to yellowish hyaline in KOH; sterigmata up to 4 µm long. ***Cheilocystidia*** (30–)30–43–58(–59) × (9–)9–11–15(–15) µm, frequent, narrowly fusiform to fusiform with obtuse apex or narrowly utriform, thin-walled, hyaline in KOH. ***Pleurocystidia*** (33–)33–43–63(–63) × (8–)8–11–13(–13) µm, infrequent, narrowly fusiform to fusiform with obtuse apex, thin-walled, hyaline in KOH. ***Hymenophoral trama*** subregular to slightly divergent, 38–82 µm wide, with subregular mediostratum 8–24 µm wide, composed of cylindrical, 4–12 µm wide hyphae, hyaline in KOH. ***Pileipellis*** an intricate trichoderm, 70–130 mm thick, made of moderately interwoven (when young) to loosely interwoven in age, thin-walled, smooth, hyaline hyphae 4–18 mm wide, branching and anastomosing at places; terminal cells 12–65 × 4–18 mm, narrowly fusiform to fusiform to broadly fusiform with slightly acuminate or obtuse apex, hyaline to yellowish pale brown in KOH. ***Pileus context*** made of strongly interwoven, thin-walled hyphae, up to 12 µm wide, hyaline in KOH. ***Stipitipellis*** arranged parallel to the surface of the stipe, composed of moderately interwoven, cylindrical, thin-walled, 3–10 µm wide hyphae, anastomosing and branching at places, sparsely scattered with groups of rising cells to clusters (up to 87 µm high) of narrowly clavate to clavate cells (21–36 × 4–9 µm), hyaline to yellowish hyaline in KOH. ***Caulocystidia*** not seen. ***Stipe context*** parallelly arranged, composed of moderately interwoven, cylindrical, thin-walled, 3–18 µm wide hyphae, hyaline to yellowish hyaline in KOH. ***Clamp connections*** not seen in any tissue.

##### Habitat and distribution.

Solitary or in small groups (up to 4 basidiomata), or fasciculate by 2 to 3 basidiomata, on sandy loam to sandy clay loam soil in open dry dipterocarp forest and dipterocarp forest dominated by Dipterocarpaceae trees namely *Anthoshorearoxburghii*, *Dipterocarpusobtusifolius*, *D.tuberculatus*, *D.intricatus*, *Pentacmesiamensis*, and *Shoreaobtusa* with or without scattered Fagaceae trees. Currently known from the type locality (Nakhon Phanom province), Sisaket and Ubon Ratchathani provinces in northeastern Thailand, and also in Chiang Mai and Chiang Rai provinces in northern Thailand.

##### Specimens examined.

Thailand, Chiang Mai Province, Muang District, Doi Suthep-Pui National Park, 18°47'39.4"N, 98°55'21.5"E, elev. 915 m, 20 July 2015, *Olivier Raspé*, OR1004 (CMUB, BKF, BR); ibid., 18°48'04.2"N, 98°55'44.3"E, elev. 775 m, 21 July 2015, *Santhiti Vadthanarat*, SV0155 (CMUB, BKF); Mae On District, 18°51'57.4"N, 99°17'22.9"E, elev. 660 m, 1 June 2015, *Olivier Raspé*, OR0915 (CMUB, BR); ibid., 18°51'57.0"N, 99°17'23.0"E, elev. 660 m, 1 June 2015, *Olivier Raspé*, OR0918 (CMUB, BR); ibid., 18°51'57.0"N, 99°17'23.0"E, elev. 660 m, 1 June 2015, *Olivier Raspé*, OR0919 (CMUB, BR); ibid., 18°52'13.0"N, 99°18'25.0"E, elev. 760 m, 15 August 2015, *Santhiti Vadthanarat*, SV0219 (CMUB, BR); ibid., 18°51'57.4"N, 99°17'22.0"E, elev. 700 m, 16 August 2015, *Santhiti Vadthanarat*, SV0225 (CMUB, BR); ibid., 18°51'57.7"N, 99°17'26.5"E, elev. 685 m, 1 June 2017, *Santhiti Vadthanarat*, SV0397 (CMUB, BR); ibid., 18°52'15.6"N, 99°18'11.5"E, elev. 800 m, 11 July 2017, *Olivier Raspé*, OR1392 (CMUB, BR); ibid., 18°52'15.6"N, 99°18'11.5"E, elev. 800 m, 11 July 2017, *Olivier Raspé*, OR1399 (CMUB, BR); ibid., 18°52'16.7"N, 99°18'13.0"E, elev. 800 m, 9 June 2021, *Santhiti Vadthanarat*, SV0512 (CMUB, BR); ibid. 18°52'7.9"N, 99°17'42.0"E, elev. 780 m, 10 June 2021, *Santhiti Vadthanarat*, SV0517 (CMUB, BR); ibid. 18°52'16.4"N, 99°17'40.5"E, elev. 820 m, 10 June 2021, *Santhiti Vadthanarat*, SV0518 (CMUB, BR); ibid. 18°52'12.0"N, 99°17'31.2"E, elev. 700 m, 10 June 2021, *Bhavesh Raghoonundon*, BR0311; ibid. 18°52'26.8"N, 99°18'15.5"E, elev. 845 m, 10 June 2021, *Bhavesh Raghoonundon*, BR0313; Chiang Rai Province, Phan District, 19°48'50.0"N, 99°51'57.0"E, elev. 730 m, 22 June 2021, *Bhavesh Raghoonundon*, BR0368; ibid. 19°48'50.0"N, 99°51'57.0"E, elev. 730 m, 22 June 2021, *Bhavesh Raghoonundon*, BR0371; Sisaket Province, Kanthararom District, Kok Yang Yai roadside market, 17 September 2016, *Santhiti Vadthanarat*, SV0345 (CMUB); Ubon Ratchathani Province, Trakan Phuet Phon District, Huay Fai, 15°32'44.3"N, 105°10'17.4"E, elev. 165 m, 28 July 2015, *Olivier Raspé*, OR1059 (CMUB, BR).

ITS sequence accession number (SV0155): PP354891.

##### Notes.

The BLAST result based on ITS sequence obtained from one of the examined specimens (voucher SV0155, GenBank accession number PP354891) was 100% identical to the holotype of *X.sisongkhramensis* (voucher BBH 48255, accession number OP462477) which was reported by Tan et al. 2022. This suggested that our collections belonged to *X.sisongkhramensis.* Morphological characters of our collections mostly fit the original description of the species. However, some variations were observed between ours and the original description as follows: Tan et al. (2022) mentioned the absence of cheilocystidia in *X.sisongkhramensis* while we could observe them in our collections; they were narrowly fusiform to fusiform with obtuse apex or narrowly utriform, thin-walled. The protologue mentioned broadly clavate to subclavate (40–60 × 8–15 μm) caulocystidia. However, in our observation only groups of rising terminal cells of shape and size similar to the caulocystidia in Tan et al. (2022), were observed. What Tan et al. (2022) considered as caulocystidia were what we described as undifferentiated terminal cells of the stipitipellis. In the species protologue, the pileipellis and stipitipellis were described as composed of thick-walled hyphae (no measurement mentioned). However, only thin-walled hyphae were observed in our collections.

**Figure 3.**
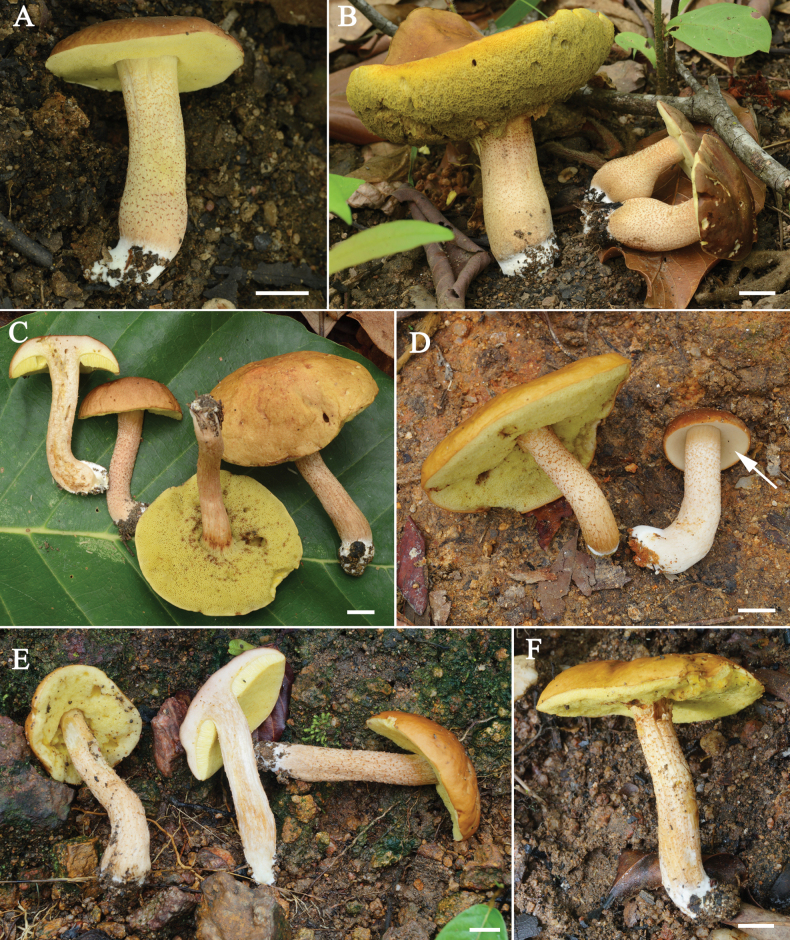
Fresh basidiomata of *Rostrupomycessisongkhramensis***A** OR0915 **B** OR0919 **C** OR1004 **D** SV0155, white pores surface in young basidioma (white arrow) **E** SV0219 **F** SV0225. Scale bars: 1 cm (**A–F**).

*Rostrupomycessisongkhramensis* is morphologically similar to *Hemileccinumduriusculum* Mei-Xiang Li, Zhu L. Yang & G. Wu, which was recently described from China. The two species share some morphological characters including basidiome size and color, scattering of granular squamules on the stipe surface, pale yellow to grayish yellow hymenophore that is depressed around the stipe apex, and unchanging color in any parts. However, *H.duriusculum* differs by its strikingly venose pileus surface, finer granular squamules on the stipe surface, and subfusiform basidiospores ornamented with irregular warts under SEM (Liu et al. 2024). *Rostrupomycessisongkhramensis* is also somewhat similar to a European *Leccinum* species originally described from Italy, *Leccinumalbostipitatum* den Bakker & Noordel., which has a similar shade of pileus color (light orange), whitish stipe covered with whitish squamules when young to reddish brown in age. However, *L.albostipitatum* can be differentiated by having an inflexed margin which exceeds the hymenophore by up to 4 mm, yellowish white to very pale brown hymenophore that becomes brownish when bruised, a clear blue discoloration of the stipe base when touched, context staining vinaceous then grayish to blackish when cut, smooth fusiform basidiospores, distribution in Europe, and association with *Populus* L. trees (den Bakker and Noordeloos 2005).

**Figure 4.**
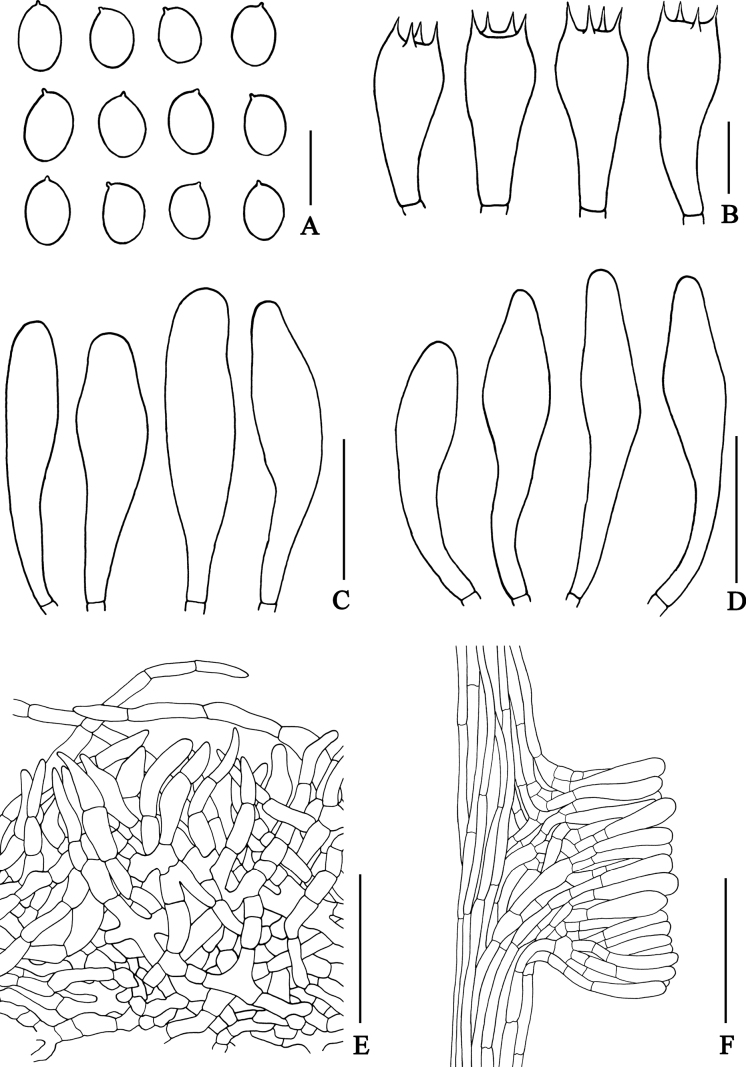
Microscopic features of *Rostrupomycessisongkhramensis***A** basidiospores **B** basidia **C** cheilocystidia **D** pleurocystidia **E** pileipellis **F** stipitipellis showing a cluster of narrowly clavate to clavate cells which slightly scattered on the stipe surface. Scale bars: 10 µm (**A–D**); 25 µm (**D–E**); 50 µm (**E–F**). All line drawings were made from SV0155.

Phylogenetically, *R.sisongkhramensis* is closely related to *Rubinosporusauriporus* Vadthanarat, Raspé & Lumyong, the only known species in the genus, which was described from the same region as *Rostrupomyces* (northern Thailand). However, it can be differentiated from *R.sisongkhramensis* by having grayish red to pastel red to reddish brown pileus; even stipe surface with scattered bright yellow to yellowish white to orange to light brown minute squamules; shorter tubes especially when young; golden yellow hymenophore; and the striking dark ruby spore print (Vadthanarat et al. 2022).

**Figure 5.**
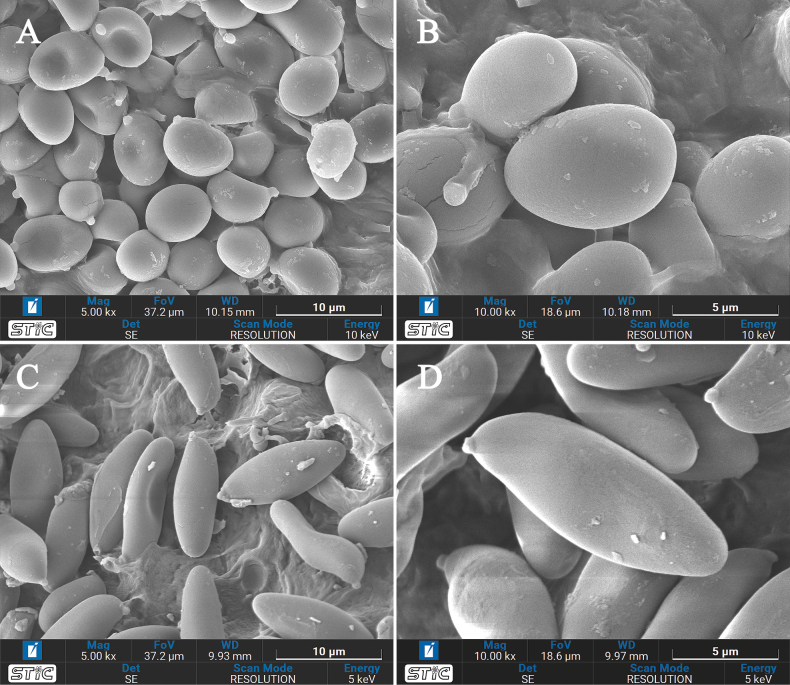
Scanning electron micrographs of basidiospores **A–B***Rostrupomycessisongkhramensis* (SV0155) **C–D***Hemileccinuminferius* (SV0282).

#### ﻿ Hemileccinum inferius

Taxon classificationFungiBoletalesBoletaceae

Vadthanarat, Raghoonundon & Raspé sp. nov.

FBB11244-EB67-5416-B51F-61EC4CBB7DE4

849063

Figs 6 , 7

##### Etymology.

“inferius” refers to the only lower part of the stipe ornamented with reticulum

##### Holotype.

Thailand, Chiang Mai Province, Muang District, Doi Suthep-Pui National Park, 18°47'52.8"N, 98°54'21.2"E, elev. 1,170 m, 1 July 2016, *Santhiti Vadthanarat*, SV0282 (holotype: CMUB, isotype: BKF, MFUB). ITS sequence accession number PP354892.

##### Diagnosis.

*Hemileccinuminferius* can be differentiated from resembling *Hemileccinum* species by a grayish red to reddish brown to dark brown, plane to sub-depressed, subrugulose to pitted pileus; and yellow to yellowish white, cylindrical with subbulbous stipe, with surface even on the upper half and subscabrous to delicately reticulate on the lower half, as well as smooth basidiospores even when observed under SEM.

##### Description.

***Basidiomata*** medium-sized. ***Pileus*** 66–68 mm in diameter, plane to sub-depressed at the centre; ***margin*** deflexed in age, elastic, slightly exceeding (up to 1 mm); ***surface*** subrugulose to pitted especially near the margin, dull, moist to slippery when wet, tomentose, grayish red (8B/C3–4) to reddish brown (8D/E4–6) to dark brown to reddish brown (7–8F4–6), unchanging when bruised; ***context*** 8–10 mm thick haft-way to the margin, firm to soft, pale yellow (1A3), slightly brown (7E5) near the pileus surface, light yellow (1A4) above the hymenium in age, unchanging when cut. ***Stipe*** 65–76 × 14–18 mm, central, terete, cylindrical with subbulbous base; ***surface*** even on the upper half then subscabrous to delicately reticulate on the lower half, dull, dry to moist, light yellow (2A4–6) to yellowish white to pale yellow (2A2–3) at the base, occasionally with reddish brown to dark brown spots (8D5–8, 8F7) at places, minutely covered with pale yellow to light brown to dark brown (2A3–4 to 7D/E4, 7F8) squamules on the upper half, slightly fibrillose following a reticulate pattern at the middle of the stipe getting less so to the base, unchanging when bruised; ***basal mycelium*** white (1A1); ***context*** solid, firm, pale yellow (2A3–5) especially in the above half near the stipe surface becoming yellowish white (2A2) to off-white at the base, unchanging when cut. ***Hymenophore*** tubulate, slightly depressed around the stipe, with slightly decurrent tooth, subventricose. ***Tubes*** 7–8 mm long half-way to the margin, yellow to grayish yellow (2A7 to 2B7) near the pileus context then olive (2E5) near the pores, separable from the pileus context, unchanging when bruised. ***Pores*** 0.3–0.8(1.2) mm wide at mid-radius, subangular to angular, even, grayish yellow (2B5), unchanging when touched, irregularly arranged; topography subregular. ***Odour*** mild fungoid. ***Taste*** mild. ***Spore print*** olive brown (4E7).

***Macrochemical reactions***: KOH, brownish orange on pileus and hymenophore, pale orange on pileus context and stipe surface, and stipe context; NH_4_OH, brownish orange with purple aura on pileus, yellowish to brownish orange with purple aura on stipe surface, yellowish to greenish or slightly blue on pileus context and stipe context.

**Figure 6.**
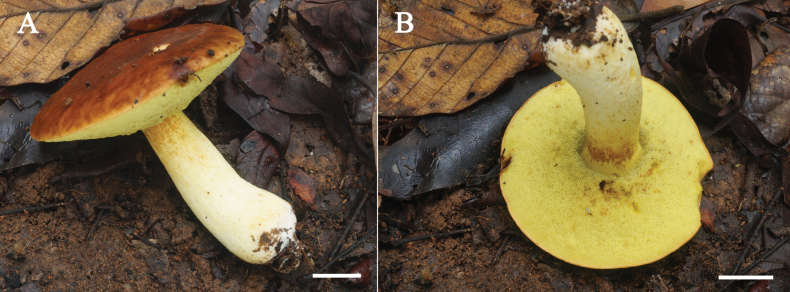
Fresh basidioma of *Hemileccinuminferius***A, B** SV0282 (holotype). Scale bars: 1 cm (**A, B**).

***Spores*** [118/2/2] (10.5–)11.4–12.9–14.6(–15.3) × (3.8–)4.2–4.8–5.6(–6.1) µm *Q* = (2.06–)2.4–2.68–3.05(–3.32). From the type (10.8–)11.5–12.7–14.2(–14.5) × (4.1–)4.3–4.8–5.5(–6.1) µm, *Q* = (2.06–)2.33–2.66–3.06(–3.1), *N* = 68, narrowly ellipsoid to subcylindrical with a slight suprahilar depression, thin-walled, smooth under light microscope and SEM (Fig. 5C–D), yellowish to brownish hyaline in water, yellowish hyaline in KOH, inamyloid. ***Basidia*** 4-spored, (23–)24–27–31(–32) × (11–)11–12–14(–14) µm, clavate without basal clamp connection, hyaline to yellowish hyaline in KOH; sterigmata up to 4 µm long. ***Cheilocystidia*** (30–)34–54–72(–72) × (7–)8–10–14(–14) µm, narrowly fusiform with elongated obtuse apex, frequent, thin-walled, hyaline to yellowish hyaline in KOH. ***Pleurocystidia*** (34–)34–51–69(–70) × (10–)10–11–13(–13) µm, frequent near the pores, narrowly fusiform with elongated obtuse apex, thin-walled, hyaline to yellowish hyaline in KOH. ***Hymenophoral trama*** slightly divergent, 62–150 µm wide composed of cylindrical, 4–12 µm wide hyphae, with subregular mediostratum 30–100 µm wide, hyaline in KOH. ***Pileipellis*** a hyphoepithelium, 80–112 μm thick, the pileipellis composed of ellipsoid to broadly ellipsoid or cylindrical, thin-walled, more or less vertically arranged, occasionally branching or anastomosing, with metablematic, elongated-cylindrical hyphae (2–4 µm wide hyphae), branching or anastomosing at places, hyaline to yellowish hyaline in KOH; terminal cells of 2 types: 1) ellipsoid to broadly ellipsoid, 8–15 × 12–20 µm; and 2) clavate to broadly clavate with obtuse apex, 10–20 × 4–7 µm. ***Pileus context*** made of moderately interwoven, ellipsoid to broadly ellipsoid, thin-walled hyphae, 10–23 µm wide, hyaline in KOH. ***Stipitipellis*** arranged parallel to the surface of the stipe (40–50 µm thick), composed of moderately interwoven, cylindrical, thin-walled, 2.5–4 µm wide hyphae, anastomosing at places, moderately scattered with groups of rising cells to clusters (50–60 µm high) of thin-walled clavate to broadly clavate cells (20–30 × 10–15 µm), hyaline to yellowish hyaline in KOH. ***Caulocystidia*** not seen. ***Stipe context*** composed of parallel, 8–22 µm wide hyphae, hyaline in KOH. ***Clamp connections*** not seen in any tissue.

**Figure 7.**
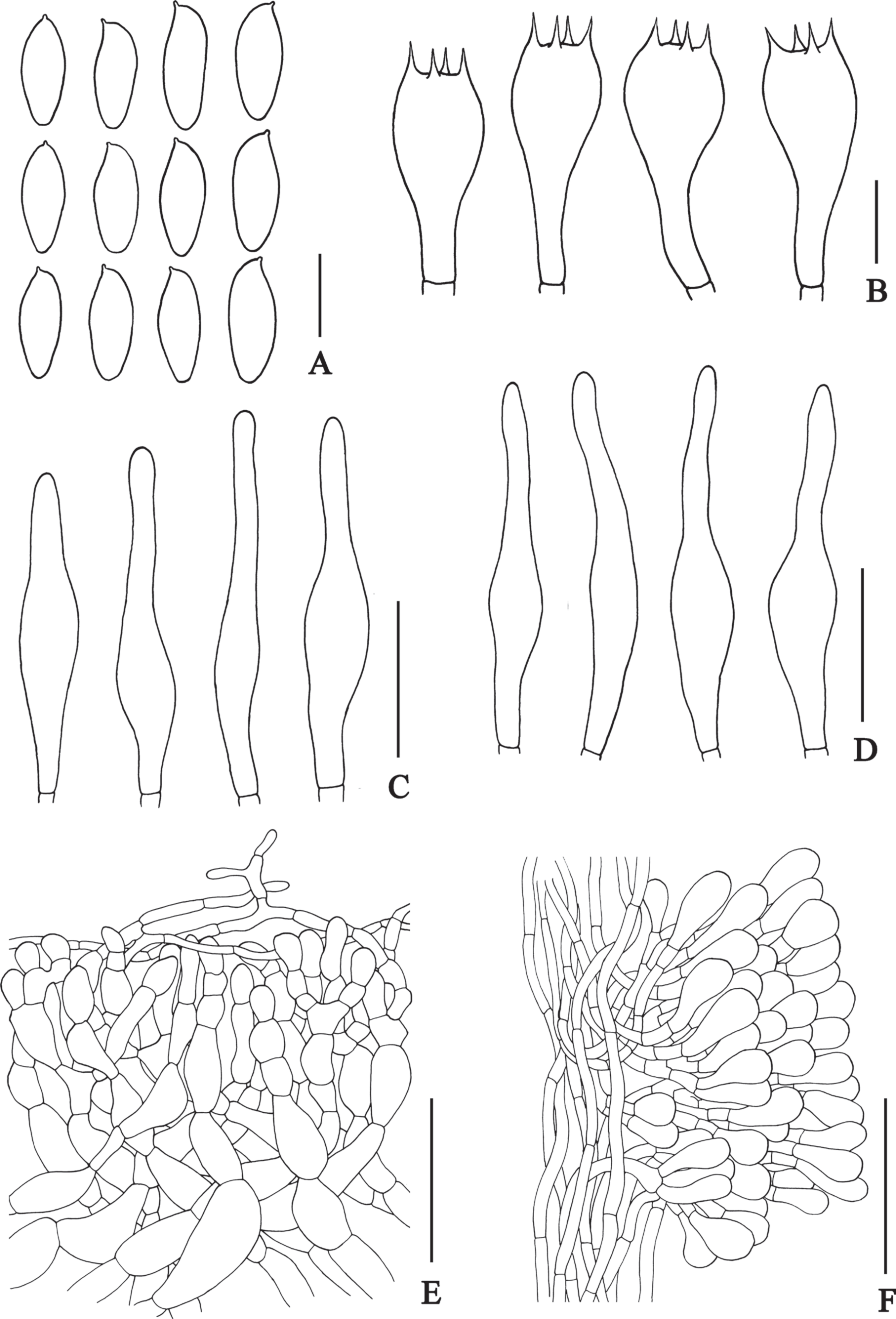
Microscopic features of *Hemileccinuminferius***A** basidiospores **B** basidia **C** cheilocystidia **D** pleurocystidia **E** pileipellis **F** stipitipellis showing a cluster of clavate to boardy clavate like cells which moderately scattered on the stipitipellis. Scale bars: 10 µm (**A–D**); 25 µm (**D–E**); 50 µm (**E–F**). All drawings were made from holotype type (SV0282).

##### Habitat and distribution.

Solitary, on loamy soil in hill evergreen forest dominated by Fagaceae scattered with a few *Dipterocarpusobtusifolius*, at 985–1,170 m elevation. Currently known from Chiang Mai Province, northern Thailand.

##### Additional specimens examined.

Thailand, Chiang Mai Province, Mae Taeng District, 19°06'59"N, 98°44'23"E, elev. 985 m, 6 June 2021, *Bhavesh Raghoonundon*, BR0260 (MFLU).

##### Notes.

*Hemileccinuminferius* is described based on collections from Thailand. The comparison of the new species with the seven known Asian species follows. *Hemileccinumalbidum* differs from *H.inferius* by gray-brown to chrome yellow to ochraceous or golden brown pileus; longer and slender stipe (up to 160 mm); shorter basidiospores (10–12.5 × 4.0–5.5 µm); occurrence at higher elevations (1,968–2,490 m; Li et al. 2021). *Hemileccinumbrevisporum* is similar in pileus color but has shorter basidiospores (9–11 × 4–5 μm); and it occurs under Fagaceae and Pinaceae, at higher elevations (1,700–2,120 m); Li et al. 2021). *Hemileccinumduriusculum* is macromorphologically quite similar, but differs by a strongly venose pileus surface, even when young, the absence of reticulum on the lower half of the stipe, as well as shorter cheilo- and pleurocystidia (Liu et al. 2024). *Hemileccinumferrugineipes* has similar pileus surface and color but can be differentiated by the apparent pale red-brown color on the lower part of the stipe; and shorter basidiospores (11.0–12.5 × 4–5 μm; Li et al. 2021). *Hemileccinumindecorum* is clearly different in having dark red to reddish brown basidiomata with mucilaginous surface densely covered with whitish to dirty white, small conical to subconical to irregularly shaped squamules; incurved margin; and yellowish hymenophore that slowly turns brownish to reddish brown when bruised (Horak 2011; Zeng et al. 2012). *Hemileccinumparvum* has smaller basidiomata (pileus 3.3–3.6 cm diam, stipe 60–97 × 4–9 mm); paler pileus (brownish to yellowish); pale yellow context that slowly turns pale blue when cut (Li et al. 2021). *Hemileccinumrugosum* has paler pileus (light orange to reddish orange); very distinctly rugose to wrinkled pileus surface; and shorter basidiospores (9–13 × 4–5 µm; Wu et al. 2016).

*Hemileccinuminferius* is also similar to an American species, *H.floridanum*, which has reddish brown to chestnut brown wrinkled and uneven pileus, whitish to pale yellow stipe, white basal mycelium, yellow hymenophore, and smooth basidiospore under both light microscope and SEM. However, the latter species is different by white context that slowly turns yellow often from the margin toward the center, longer basidiospores (10–17 × 4.5–6 μm), likely forms association with oak in northern America (Farid et al. 2021).

Phylogenetically, *H.inferius* was most closely related to *H.hortonii*, *H.rugosum*, and an undescribed specimen (voucher HKAS 53421) from China. *Hemileccinumhortonii*, an American species, can easily be distinguished by its conspicuously pitted pileus, smooth to lightly pruinose stipe that sometimes has delicate reticulation on the upper half, pores that occasionally turn blue on when touched, and slightly longer and narrower basidiospores (12–15 × 3.5–4.5; Kuo and Ortiz-Santana 2020; Farid et al. 2021). For morphological comparison with *H.rugosum* see the above paragraph.

## ﻿Discussion

In this study, the morphological and phylogenetic evidence highly supported establishing *Rostrupomyces* as a new genus of Boletaceae to accommodate *Xerocomussisongkhramensis*. The most important morphological characters used to differentiate the new genus from other Boletaceae genera are: subscabrous stipe surface with scattered granulose squamules; hymenophore that is white in young basidiomes and becomes yellow in age; yellowish brown spore print; and broadly ellipsoid to ellipsoid basidiospores with smooth surface.

The character of subscabrous to scabrous stipe surface dotted with scattered granulose squamules is also present in other Boletaceae genera such as *Hemileccinum* (see in notes under *Rostrupomyces*), *Leccinum* Gray, *Leccinellum* Bresinsky & Manfr. Binde, *Rugiboletus* G. Wu & Zhu L. Yang, and *Sutorius* Halling, Nuhn & N.A. Fechner. *Leccinum* can be separated from *Rostrupomyces* by having a white to pallid to light brown hymenophore while *Leccinellum* has yellow hymenophore similar to *Rostrupomyces*. However, both genera are different from *Rostrupomyces* by a more or less pronounced color change of hymenophore, stipe surface, and/or context, which can stain red, brown, yellow, or occasionally blue when bruised. *Leccinellum* and *Leccinum* produce boletoid basidiospores which are also different from *Rostrupomyces*. Moreover, they are phylogenetically distant and placed in another subfamily, the Leccinoideae (den Bakker and Noordeloos 2005; Wu et al. 2016; Xue et al. 2019; Meng et al. 2021). *Rugiboletus* differs from *Rostrupomyces* by its strongly wrinkled pileus (especially when young), yellow or brown or reddish brown hymenophore that is unchanging or turns bluish when bruised, subfusiform basidiospores, and phylogenetically distant and placed in *Pulveroboletus* group (Wu et al. 2015; Kuo and Ortiz-Santana 2020). *Sutorius* Halling, Nuhn & N.A. Fechner, is different in having chocolate to reddish brown or purplish brown basidiomata, grayish or reddish brown or brownish orange hymenophore, context always with scattered reddish or violet or dark brown encrustations that are visible with the naked eye, reddish brown spore deposit, and narrowly ellipsoid to subcylindrical basidiospores (Halling et al. 2012; Vadthanarat et al. 2021). Like *Rugiboletus*, *Sutorius* is phylogenetically distant from *Rostrupomyces*, belonging to the *Pulveroboletus* group (Vadthanarat et al. 2021).

Xerocomoideae genera other than *Rostrupomyces* also produce smooth basidiospores, including *Amylotrama*, *Aureoboletus*, *Alessioporus*, *Pulchroboletus*, *Rubinosporus*, and *Veloboletus*. Moreover, while most *Xerocomus* and *Phylloporus* species produce basidiospores with bacillate surface, a few species produce smooth basidiospores (Neves and Halling 2010; Wu et. al. 2016; Chuankid et al. 2019). However, only *Amylotrama* and *Rubinosporus* present the same shape of basidiospore as *Rostrupomyces*, whereas the others produce more or less oblong to ellipsoid to fusiform basidiospores (Gelardi et al. 2014; Wu et al. 2016; Farid et al. 2017; Frank et al. 2017; Zhang et al. 2019; Crous et al. 2020; Lebel et al. 2022; Vadthanarat et al. 2022). *Amylotrama* comprises two species from Australia, which are completely different from *Rostrupomyces* by their sequestrate basidiomata (Lebel et al. 2022). *Rubinosporus*, differs by having a strikingly thin hymenophore, especially when young; golden yellow hymenophore; and dark ruby spore print (Vadthanarat et al. 2022). *Aureoboletus* differs by the pileus usually having a viscid surface especially when moist; and golden yellow hymenophore (Wu et al. 201; Zhang et al. 2019). *Alessioporus* is different by its reticulated stipe occasionally with a granular ring-like zone in the middle or lower half of the stipe, golden yellow hymenophore; blue staining of the stipe surface, hymenophore, and context; and distribution in Mediterranean Italy and subtropical USA (Gelardi et al. 2014; Frank et al. 2017). *Pulchroboletus* differs by the stipe surface with scattered red to reddish brown, occasionally with reticulum or longitudinal striations, and with a pseudo-annulus; golden yellow hymenophore; intense blue staining of the hymenophore and context; and occurrence only in Mediterranean Europe and tropical to subtropical America (Gelardi et al. 2014; Farid et al. 2017). The only *Veloboletus* species, is different by its basidiomata with a distinctive universal veil; blue staining of the pileus, stipe, hymenophore, and context, and distribution in Australia (Crous et al. 2020).

Tan et al. (2022) phylogeny was based on ITS and LSU sequences of only *Xerocomus* spp., and *Phylloporus* as outgroup, which resulted in the clustering of *X.sisongkhramensis* in *Xerocomus*. However, our phylogeny based on multiple protein-coding genes (*atp*6, *cox*3, *tef*1, and *rpb*2) and on a much wider taxon sampling of Boletaceae resolved *X.sisongkhramensis* in subfamily Xerocomoideae indeed, but distant from other *Xerocomus* specie*s.* Keeping *X.sisongkhramensis* would have rendered the genus polyphyletic. The erection of the new genus *Rostrupomyces*, which can also be morphologically separated from *Xerocomus*, was therefore necessary.

In the phylogeny, *Rostrupomyces* appeared sister to another monotypic genus, *Rubinosporus* (morphological comparison see in notes under *Rostrupomycessisongkhramensis*). The two genera can be differentiated mainly by the spore print color, and color of hymenophore, two characters that do not vary between species in the same genus in Boletaceae. The characters have been primarily used to differentiate many genera in Boletaceae e.g., *Sutorius*, *Cacaoporus*, *Hourangia*, *Baorangia* (Halling et al. 2012; Wu et al. 2015; Zhu et al. 2015; Vadthanarat et al. 2019b). Additional morphological characters, including pileus color and stipe surface, could be useful to separate them. However, both genera so far comprise only a single species and the pileus color and stipe surface are found to be variable between species within the same genus. For example, in *Boletus* L. and *Tylopilus* P. Karst. the pileus color is variable from white, yellow, brown, orange, green, gray, and purple, and the stipe surface from even to reticulate to strongly reticulate (e.g., Cui et al. 2015; Wu et al. 2016; Li and Yang 2021). Hence, if more species in either of those two genera are described, the comparison between the two genera might need updating.

*Rostrupomyces* has been found so far on sandy loam to sandy clay loam soils at elevations lower than 1,000 m (165 to 915 m), in open dry dipterocarp and dipterocarp forest mainly dominated by ectomycorrhizal trees in family Dipterocarpaceae genera *Anthoshorea* (*A.roxburghii*), *Dipterocarpus* (*D.obtusifolius*, *D.tuberculatus*, *D.intricatus*), *Pentacme* (*P.siamensis*), and *Shorea* (*S.obtusa*), with scattered Fagaceae trees. In Thailand the Dipterocarpaceae tree species are mainly distributed in lowland (<800 m) to mid-elevation forests (800–1,200 m) whereas Fagaceae trees are mostly distribute in mid-elevation to highland forests (>1,200 m) (Gardner et al. 2007). During our surveys on the diversity of Boletaceae in Thailand, no *Rostrupomyces* collection was found in the forests above 1,000 m, where no *Anthoshorea*, *Dipterocarpus*, *Pentacme*, or *Shorea* trees were observed or mentioned as occurring. This suggests that the distribution of *Rostrupomyces* depends on the distribution of the mentioned tree genera, and they are inferred as the associated tree hosts of *Rostrupomyces*. However, a more detailed study is needed to confirm the specificity of its relationship with ectomycorrhizal hosts.

In this study, some specimens of *Rostrupomycessisongkhramensis* were collected from community forests and the species was found to be consumed by local people, in Ubon Ratchathani and Sisaket provinces in lower northeastern Thailand. It is found on sale on roadsides and local markets, along with other Boletaceae in genera such as *Baorangia*, *Boletus*, *Boletellus*, *Heimioporus*, *Retiboletus*, *Sutorius*, and *Tylopilus*. The species is called “Hed Phueng Waan” in which the words “Hed Phueng” refer to bolete and “Waan” means sweet. It can also be called “Hed Phueng Kaw” in which the words “Kaw” means rice. The same local names are also applied to the other bolete species that are mostly white and have sweet taste after cooking such as *Boletus* spp. In this region a local name can be used for different mushroom species which present similarly striking morphological characters. Conversely, one mushroom species may have more than one local name. *Rostrupomycessisongkhramensis* is also found in the northern parts of Thailand in Chiang Rai and Chiang Mai provinces. However, during our survey in the region, the species has never been found being collected or on sale for consumption by the locals. The protologue of this species (collections from upper northeastern Thailand) did not mention the edibility (Tan et al. 2022).

To date, 15 *Hemileccinum* species have been described worldwide, among which eight are originally from Asia (China: *H.albidum*, *H.brevisporum*, *H.duriusculum*, *H.ferrugineipes*, *H.parvum*, *H.rugosum*; Singapore: *H.indecorum*; and Thailand: *H.inferius*), two species from France in Europe (*H.depilatum* and *H.impolitum*), four species from North America (*H.floridanum*, *H.hortonii*, *H.rubropunctum*, and *H.subglabripes*), and a single species, *H.brunneotomentosum*, from Belize in Central America (Šutara 2008; Halling et al. 2015; Wu et al. 2016; Kuo and Ortiz-Santana 2020; Nitson and Frank 2020; Farid et al. 2021; Li et al. 2021; Liu et al. 2024). Three *Hemileccinum* species have been previously reported to occur in Thailand, namely *H.depilatum* (reported as *Boletusdepilatus* Redeuilh), *H.impolitum* (reported as *B.impolitus* Fr.), and *H.indecorum* (Chandrasrikul et al. 2011; Vadthanarat et al. 2019b). The first two species were originally described from France and were then reported from Thailand based on morphological identification only. As we know that the distribution of Boletaceae species depends on the distribution of their hosts, the ecology and host specificity are important characters in distinguishing species in Boletaceae (den Bakker et al. 2004; Dentinger et al. 2010; Cui et al. 2015; Loizides et al. 2019; Gelardi 2020). It is therefore doubtful that European species are also present in Southeast Asia where the forests are dominated by different tree species or families. Unfortunately, no specimens associated with the reports of occurrence in Thailand are available for molecular analysis to compare with European specimens. Moreover, molecular analysis of several *Hemileccinum* specimens obtained in our study showed none of them belong to those European species. It is therefore reasonable to assume that the identifications of the Thai collections as *H.depilatum* and *H.impolitum* were not correct. The other recorded species, *H.indecorum* was originally described from Singapore in Southeast Asia (Horak 2011). Specimens collected from Thailand were identified based on both molecular and morphological evidences (Vadthanarat et al. 2019b). However, the full morphological description of this Thai collection has not yet been published. In the future, more detail on the species and more records of *Hemileccinum* will be reported.

Basidiospores with tiny warts and pinholes (when observed under SEM) are typical of *Hemileccinum*. However, a few *Hemileccinum* species produce basidiospores with smooth surface, including the new species (Kuo and Ortiz-Santana et al. 2020; Farid et al. 2021). This kind of exception is also found in other Xerocomoideae genera i.e., in *Phylloporus* and *Xerocomus*, In the latter two genera, most of the species produce basidiospores with bacillate surfaces, but a few produce smooth basidiospores (Neves and Halling 2010; Wu et. al. 2016; Chuankid et al. 2019).

A total of 39 new taxa (4 new genera and 35 new species), including those introduced in this paper, have been originally described from Thailand (Rostrup 1902; Yang et al. 2006; Desjardin et al. 2009; Choeyklin et al. 2012; Neves et al. 2012; Halling et al. 2014; Raspé et al. 2016; Vadthanarat et al. 2018; Chuankid et al. 2019; Phookamsak et al. 2019; Vadthanarat et al. 2019a, 2019b, 2020; Chuankid et al. 2021; Raghoonundon et al. 2021; Vadthanarat et al. 2021; Tan et al. 2022; Vadthanarat et al. 2022; This study). Our study on the diversity of Boletaceae in Thailand is still ongoing and is needed to uncover more new taxa and new distribution records for Thailand.

## Supplementary Material

XML Treatment for Rostrupomyces

XML Treatment for Rostrupomyces sisongkhramensis

XML Treatment for Hemileccinum inferius

## ﻿Additional information

### Author ORCIDs

Santhiti Vadthanarat https://orcid.org/0000-0002-9035-0375

Bhavesh Raghoonundon https://orcid.org/0000-0001-6671-2404

Olivier Raspé https://orcid.org/0000-0002-8426-2133

### Data availability

All of the data that support the findings of this study are available in the main text.
