# ﻿Corrigendum: Vadthanarat S, Raghoonundon B, Lumyong S, Raspé O (2024) *Rostrupomyces*, a new genus to accommodate *Xerocomussisongkhramensis*, and a new *Hemileccinum* species (Xerocomoideae, Boletaceae) from Thailand. MycoKeys 103: 129–165. https://doi.org/10.3897/mycokeys.103.107935

**DOI:** 10.3897/mycokeys.107.132226

**Published:** 2024-08-13

**Authors:** Santhiti Vadthanarat, Bhavesh Raghoonundon, Saisamorn Lumyong, Olivier Raspé

**Affiliations:** 1 School of Science, Mae Fah Luang University, Chiang Rai, 57100, Thailand; 2 Biology Department, Faculty of Science, Chiang Mai University, Chiang Rai, Thailand; 3 Department of Biology, Faculty of Science, Chiang Mai University, Chiang Mai, 50200, Thailand; 4 Research Center of Microbial Diversity and Sustainable Utilization, Faculty of Science, Chiang Mai University, Chiang Mai, 50200, Thailand; 5 Academy of Science, The Royal Society of Thailand, Bangkok, Thailand; 6 Meise Botanic Garden, Nieuwelaan 38, 1860 Meise, Belgium; 7 Service Général de l’Enseignement Supérieur et de la Recherche Scientifique, Fédération Wallonie-Bruxelles, Brussels, Belgium

It was kindly brought to our attention that the names *Rostrupomyces* and *Rostrupomycessisongkhramensis* were not valid because incomplete designation of the type species of the former name and of the basionym of the latter name (Shenzhen code: Art. 40.1, see Arts 40.3 and Arts 6.3, 12.1; and Art. 41.5; Turland et al. 2018). Therefore, we would like to properly typify the genus and cite the basionym of the new combination, as follows.

***Rostrupomyces* Vadthanarat & Raspé, gen. nov.**

**Type species.***Xerocomussisongkhramensis* Khamsuntorn, Pinruan & Luangsa-ard Persoonia 49: 295 (2022).

***Rostrupomycessisongkhramensis* (Khamsuntorn, Pinruan & Luangsa-ard) Vadthanarat, Raghoonundon & Raspé, comb. nov.**

**Basionym.***Xerocomussisongkhramensis* Khamsuntorn, Pinruan & Luangsa-ard, Persoonia 49: 295 (2022).

## ﻿Additional information

### Author ORCIDs

Santhiti Vadthanarat https://orcid.org/0000-0002-9035-0375

Bhavesh Raghoonundon https://orcid.org/0000-0001-6671-2404

Saisamorn Lumyong https://orcid.org/0000-0002-6485-414X

Olivier Raspé https://orcid.org/0000-0002-8426-2133

### Data availability

All of the data that support the findings of this study are available in the main text.
